# Specific macrophage populations promote both cardiac scar deposition and subsequent resolution in adult zebrafish

**DOI:** 10.1093/cvr/cvz221

**Published:** 2019-08-16

**Authors:** Laura Bevan, Zhi Wei Lim, Byrappa Venkatesh, Paul R Riley, Paul Martin, Rebecca J Richardson

**Affiliations:** 1 School of Physiology, Pharmacology and Neuroscience, University of Bristol, Biomedical Sciences Building, University Walk, Bristol BS8 1TD, UK; 2 Comparative and Medical Genomics Laboratory, Institute of Molecular and Cell Biology, A*STAR, 61 Biopolis Drive, Singapore 138673, Singapore; 3 Department of Paediatrics, Yong Loo Lin School of Medicine, National University of Singapore, Singapore 119228, Singapore; 4 Department of Physiology, Anatomy and Genetics, University of Oxford, Sherrington Building, South Parks Road, Oxford OX1 3PT, UK; 5 School of Biochemistry, University of Bristol, Bristol, UK

**Keywords:** Zebrafish, Regeneration, Scarring, Heart failure, Inflammation

## Abstract

**Aims:**

A robust inflammatory response to tissue injury is a necessary part of the repair process but the deposition of scar tissue is a direct downstream consequence of this response in many tissues including the heart. Adult zebrafish not only possess the capacity to regenerate lost cardiomyocytes but also to remodel and resolve an extracellular scar within tissues such as the heart, but this scar resolution process remains poorly understood. This study aims to characterize the scarring and inflammatory responses to cardiac damage in adult zebrafish in full and investigate the role of different inflammatory subsets specifically in scarring and scar removal.

**Methods and results:**

Using stable transgenic lines, whole organ imaging and genetic and pharmacological interventions, we demonstrate that multiple inflammatory cell lineages respond to cardiac injury in adult zebrafish. In particular, macrophage subsets (*tnfα+* and *tnfα*−*)* play prominent roles with manipulation of different phenotypes suggesting that pro-inflammatory (*tnfα+*) macrophages promote scar deposition following cardiac injury whereas *tnfα*− macrophages facilitate scar removal during regeneration. Detailed analysis of these specific macrophage subsets reveals crucial roles for Csf1ra in promoting pro-inflammatory macrophage-mediated scar deposition. Additionally, the multifunctional cytokine Osteopontin (Opn) (*spp1*) is important for initial scar deposition but also for resolution of the inflammatory response and in late-stage ventricular collagen remodelling.

**Conclusions:**

This study demonstrates the importance of a correctly balanced inflammatory response to facilitate scar deposition during repair but also to allow subsequent scar resolution, and full cardiac regeneration, to occur. We have identified Opn as having both pro-fibrotic but also potentially pro-regenerative roles in the adult zebrafish heart, driving Collagen deposition but also controlling inflammatory cell resolution.

## Graphical Abstract

**Graphical Abstract:**
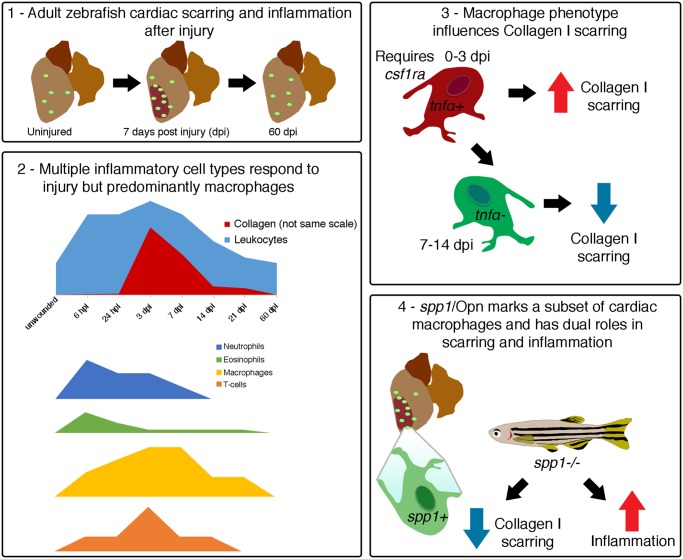

## 1. Introduction

Complete tissue regeneration of multiple organs, including the heart, has been demonstrated in several vertebrate model systems such as the zebrafish.^1–5^ Mammals retain some regenerative capacity during early neonatal periods but later lose this ability.^6^^,^^7^ Instead, tissue injury in adult mammals, including ischaemic events such as a myocardial infarction, results in adverse tissue remodelling and irreversible scarring, producing a non-functional region of cardiac tissue. The inflammatory response to injury is necessary for correct and timely repair but also leads to activation of fibroblasts/stromal cells and deposition of scar collagen,^6^^,^^8^^,^^9^ limiting cardiac function and contributing to the progression of heart failure in patients.^10^

Multiple different immune cells are recruited to the ventricular myocardium following ischaemic injury in adult mammals.^11^^,^^12^ Macrophages play critical roles in tissue repair and scarring, but recent reports have suggested a, somewhat contradictory, role for macrophage subsets in complete regeneration of the neonatal mouse heart.^13^^,^^14^ Indeed, macrophage depletion at different time-points post-injury in a model of liver fibrosis can have dramatically different effects on fibrosis/scarring vs. regeneration.^15^ Additionally, recent studies in regenerative adult zebrafish and salamanders also suggest vital roles for macrophages in tissue regeneration with global reduction of numbers via clodronate liposome treatment resulting in impaired cardiac and limb regeneration.^16–19^ Therefore, there remains some controversy over the precise role of the inflammatory response in tissue regeneration and the factors that promote regeneration over fibrotic repair are not fully elucidated. Macrophages exist as a spectrum of different activation states that are controlled by a range of factors including transcriptional and epigenetic regulation, developmental pathways, and the local tissue microenvironment, which are incompletely understood.^20–22^ During the injury response, the appropriate balance of these activation states is thought to be vital for optimal wound healing and could be harnessed to promote regeneration.^23^^,^^24^

Here, we describe the timing, extent, and spatial distribution of the full inflammatory and scarring response to cardiac cryoinjury in a regenerative adult zebrafish model with a strong early recruitment of neutrophils followed by an influx of macrophages. Further investigation reveals two waves of macrophage populations, *tnfα+* and *tnfα*− that predominate at different time-points post-injury and manipulations of each of these populations result in dramatic impairment of scar deposition and scar resolution, respectively. Additionally, we investigate the role of Osteopontin (Opn), a multifunctional glycoprotein expressed by fibroblasts and inflammatory cells and a potent fibrotic factor in mammals,^25–27^ in the response to cardiac injury. Importantly, we have identified bifunctional roles for Opn in both initial scar collagen deposition, similar to mammals, and subsequently as a regulator of macrophage phenotype driving scar and inflammatory cell resolution in our regenerative model.

## 2. Materials and methods

### 2.1 Zebrafish lines and cardiac injury

Details of transgenic lines and cardiac cryoinjury can be found in the Supplementary Material online. Animals were anaesthetized via immersion in 0.13% MS-222 (Sigma; A5040) in aquarium facility water for all procedures, once, for a maximum of 5 min. Animals were euthanized via immersion in an overdose of anaesthetic.

### 2.2 Immunofluorescence analysis, histology, *in situ* hybridization and imaging

Standard protocols were used for immunostaining, histology, and imaging of stable transgenic fluorescence. Details and antibodies used can be found in the Supplementary Material online. *In situ* hybridization was performed using a commercial *spp1* probe (Dr-spp1; catalog no. 409501) and RNAscope^®^ technology (ACD, USA) on 4% PFA fixed TgBAC(*spp1:mCherry*) larvae and adult hearts following manufacturers protocols. Following *in situ*, samples were labelled with an anti-RFP (1:250; MBL Life science; PM005) antibody using methods described in the Supplementary Material online.

### 2.3 Clodronate liposomes and drug treatments

Anaesthetized fish were intraperitoneally (IP) injected the day before cryoinjury (regime A) or at 3 days post-injury (dpi) (regime B) with 10 μL liposomes containing either clodronate or phosphate buffered saline (PBS) (10 mg/mL; FormuMax Sientific, Inc.; F70101C-A). For alteration of macrophage phenotype, anaesthetized zebrafish were IP injected with lipopolysaccharides (LPS) (5 μg in 10 μL PBS; Sigma; L2630) or recombinant zebrafish IL10 (10 μL of 6 M in PBS; Kingfisher Biotech/Cambridge Bioscience; RP1023Z) immediately after cardiac cryoinjury.

### 2.4 FACS

Ventricles of wildtype Tg(*mpeg1:mCherry*) were collected into PBS containing 10 mM HEPES, 30 mM taurine, and 5.5 mM glucose and cells dissociated by the addition of 0.25% trypsin, 12.5 M CaCl_2_, and 5 mg/mL Collagenase II (Worthington Biochemical Corp; LS004176). Dissociated cells were suspended in Leibovitz medium L-15 containing 0.3 mM glutamine (GIBCO), 0.8 mM CaCl_2_, Pen 50 U/mL/Strep 0.05 mg/mL, and 2% FCS. *Mpeg1+* macrophages were sorted for mCherry expression on a BD Biosciences InFlux high-speed fluorescence activated cell sorter (San Jose, CA, USA).

### 2.5 Generation of TgBAC(*spp1:mCherry*) ^SN:^^374^ and *spp1*^−/−^ fish

To generate the TgBAC(*spp1:mCherry*)^SN:^^374^ reporter line, BAC recombination was performed as described.^28^ A BAC covering the full *spp1* coding sequence was obtained [CH73-213K3; BACPAC Resources Centre (BPRC)] and the following primers were used to insert mCherry downstream of the *spp1* start site (5′–3′): forward, tttagtttttctctctctgtttctcttctgttttagaatattttgcacacACCATGGTGAGCAAGGGCGAGGAG; reverse, ggtacacagaagactgtggcgacgaggagtgttaaaacaataatagatttTCAGAAGAACTCGTCAAGAAGGCG (lowercase indicates the BAC homology arms and uppercase indicates the mCherry cassette). To generate a *spp1* mutant line a TALEN site was designed to target exon 7 of zebrafish *spp1*. TALEN plasmids were synthesized by ToolGen Genomics Toolmaker (Seoul, South Korea). The target site sequence was TACCACCATCATCCCAGTCACAGTCGATCCCACGCTGGGTCCCATTATCAACA, where TACCACCATCATCCCAGTCA represents the left TALEN and GCTGGGTCCCATTATCAACA represents the right. The red highlighted residue is deleted in the C331del line and the blue highlighted residues are deleted in the CGAT327-330del line. The TALEN plasmids were linearized, transcribed individually into TALEN mRNA using mMESSAGE mMACHINE T7 ULTRA kit (Ambion). Equal amounts (2 nL) of left and right TALEN mRNA were microinjected together into single-cell zebrafish embryos.

### 2.6 Statistics

In all cases *n* numbers refer to biological replicates. All experiments were repeated at least twice. Raw data recording and analysis was conducted using GraphPad Prism6/7. Power calculations (90% power at 5% significance) indicate that six animals are adequate for each parameter for statistical significance. This is also the number of fish used per time-point by others working on repair of the heart.^2^ Due to the variability of biological replicates we found that increased numbers were sometimes required to clarify findings. In some cases, fewer biological replicates were used whenever possible. For quantification of the inflammatory and scarring responses all heart regions beyond the ventricle (atrium, bulbous arteriosus, etc., including the heart valves) were manually removed from the image (×10 objective) in ImageJ. For quantification of the scarring response the percentage of the ventricle positive for Collagen I was calculated by thresholding the fluorescent signal of the whole ventricle (DAPI) and the Collagen I alone using ImageJ. The results are presented as the fold change compared to unwounded hearts. For quantification of the inflammatory response a threshold was applied to images of the whole ventricle (either sections or whole mount images) and the number of positive cells determined in ImageJ. For quantification of the scarring and inflammatory responses in sections, an average of three sections through the injury site were taken for each heart, to ensure a representative value was determined. The numbers of eosinophils and T-cells in whole mount images were counted manually due to the dim fluorescent signal. To determine the percentage of pro-inflammatory macrophages, images were taken just of the injury site (×25 objective) and the numbers of *mpeg1+* cells co-expressing *tnfα* determined manually. All GFP+ cells, regardless of intensity, were counted and all cells with clear outlines were counted in each image (typically 250–600 cells). Counts were taken from maximum projections of *z*-stacks to ensure maximum numbers of cells were analysed. Only cells where clear co-localization (e.g. by cell shape/outline) of both fluorophores was apparent were counted to prevent errors regarding overlap of cells. For all data sets a ROUT outlier test was performed and any significant outliers [*Q* (maximum false discovery) = 1%] were removed. Statistical significance was determined via non-parametric Mann–Whitney or Kruskal–Wallis/Dunn’s multiple comparison tests (details provided in figure legends). In all cases error bars represent SD. Indicated significance = *P < 0.05; **P < 0.01; ***P < 0.005; ****P < 0.001.

### 2.7 Study approval

All experiments were conducted with approval from the local ethical review committee at the University of Bristol and in accordance with UK Home Office regulations.

## 3. Results

### 3.1 Characterization of the scarring and total inflammatory responses to cardiac injury

**Figure 1:**
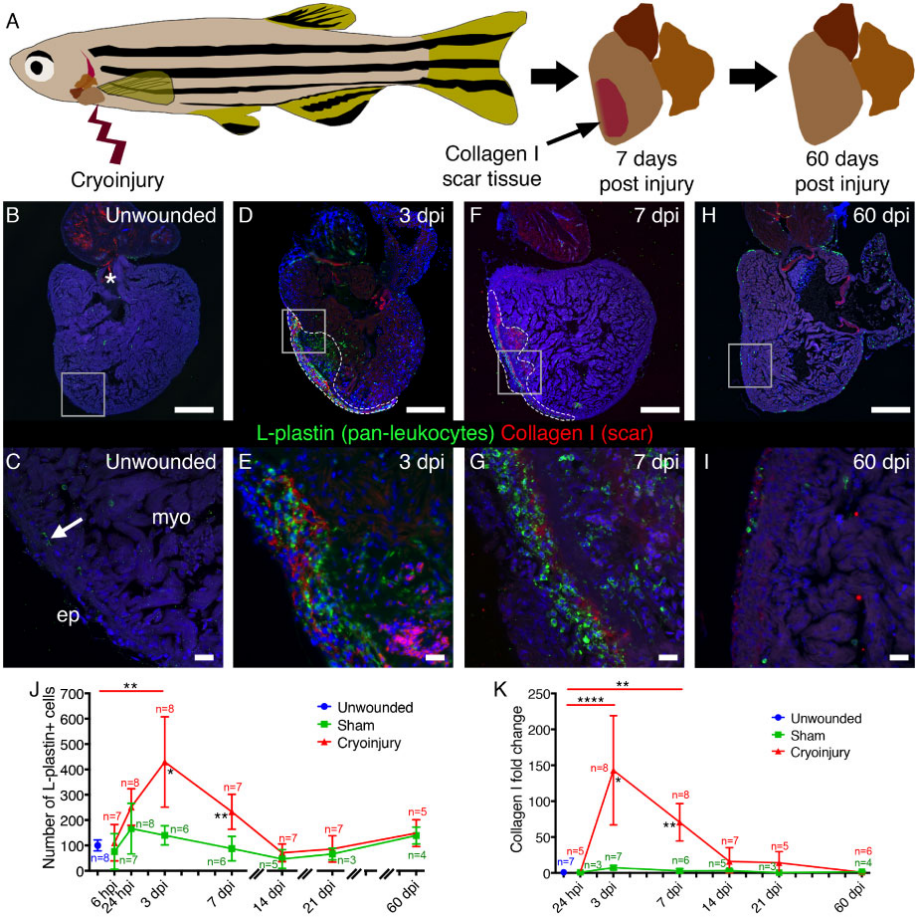
Analysis of the inflammatory and scarring response to cardiac cryoinjury. (*A*) Schematic showing the process of cardiac scarring and scar resolution in adult zebrafish following cryoinjury to the ventricle. (*B–I*) Representative images of immunofluorescence analysis of the pan-leucocyte marker, L-plastin, and the major scar matrix component, Collagen I, in cardiac tissues of unwounded and cryoinjured fish. The boxes in *B, D, F,* and *H* demark the approximate position of *C, E, G,* and *I*, respectively. The dashed lines in *D* and *F* demark the extent of the injured region. (*B* and *C*) In unwounded adult zebrafish hearts a sparse population of L-plastin+ leucocytes (arrowed in *C*) is observed. Some Collagen I expression is observed at low levels around the edge of the ventricle and at much higher levels in the cardiac valves (asterisks in *B*). (*D–G*) At 3 (*D* and *E*) and 7 days post-injury (dpi) (*F* and *G*), significant inflammatory and scarring responses are observed in the ventricle. (*H* and *I*) At 60 dpi both responses have resolved and the ventricle resembles that of unwounded hearts. (*J* and *K*) Quantification of the number of L-plastin+ cells (*J*) and the fold change of the percentage of the ventricle positive for Collagen I staining (*K*) within sections through the heart for the injury type and time-points indicated. Three sections through the injury area were quantified and an average taken for each fish. For each time-point and injury type *n* numbers are indicated. For statistical analyses in *J* and *K*, analysis of differences between sham and cryoinjuries were performed with a Kruskal–Wallis test at each time-point. Significant differences between sham and cryoinjury are shown beneath the cryoinjury line for relevant time-points. Kruskal–Wallis/Dunn’s multiple comparisons tests were used to analyse all data against unwounded (significance bars shown above graphs). All other values were not significant. Ep, epicardium; myo, myocardium. Scale bars: *B, D, F, H* = 250 μm, *C, E, G, I* = 20 μm.

Cryoinjury to the adult zebrafish ventricle leads to repair with transient scarring that resolves over time (*Figure 1A*^3^). Mammalian studies have suggested a critical role for the inflammatory response in the formation of an extracellular scar^8^^,^^9^ but the precise involvement of immune cell lineages in scar deposition/resolution in a regenerative model has not been fully elucidated. We first determined the timing of the total inflammatory and scarring response to sham and cryoinjuries in comparison to unwounded tissue (*Figure 1B–K*). In unwounded hearts a tissue resident population of L-plastin+ immune cells resided predominantly around the edge of the ventricle (*Figure 1B* and *C*). L-plastin is a pan-leucocyte marker in zebrafish and mammals.^29^^,^^30^

The scarring response was assessed by immunofluorescence analysis and subsequent quantification of Collagen I, suggested to be the major scar component.^31^ Collagen I was observed at low levels beneath the epicardium and at high levels in the cardiac valves in unwounded hearts (*Figure 1B* and *C*). Following sham injury, no significant inflammatory or scarring responses were observed (*Figure 1J* and *K*). By contrast, cryoinjury elicited a significant inflammatory response at 3 dpi, and a significant scarring response at 3 and 7 dpi (*Figure 1D–G, J*, and *K*). Inflammatory cell influx and scarring were significantly elevated in cryoinjured hearts compared to sham injury at 3 and 7 dpi (*Figure 1J* and *K*). At 3 dpi, L-plastin+ cells are distributed throughout the myocardium, accumulating close to the injury site where considerable Collagen I deposition is observed (*Figure 1D* and *E*). By 7 dpi, L-plastin+ cells are restricted almost exclusively to the injury site where significant Collagen I is still detected (*Figure 1F* and *G*). At 14, 21, and 60 dpi both the inflammatory and scarring responses are reduced (*Figure 1H–K*). Indeed, at 60 dpi the heart resembles that of an unwounded fish with the deposited Collagen I completely resolved (*Figure 1H* and *I*).

Comparison of Collagen I and acid fuchsin orange G (AFOG) histological staining, which is commonly used to label scar collagen in injured hearts, on adjacent slides revealed similar distribution of collagens with both methods, particularly at early time-points (Supplementary material online, *Figure S1*). Collagen I staining generally gave a clearer result that was easier to quantify and less variable between different experiments. Interestingly, at 60 dpi (when scar tissue was only present in a few fish) Collagen I appears more restricted than collagen labelled with AFOG (Supplementary material online, *Figure S1E and F*) suggesting a different composition of these rare late scars, supporting previous results suggesting that interstitial fibroblasts express collagens other than Collagen I at later time-points.^32^ These preliminary examinations provide an accurate description of the time-line of repair and regeneration in relation to scarring and the total inflammatory response in the adult zebrafish heart.

### 3.2 Multiple inflammatory cell lineages respond to cardiac injury

**Figure 2:**
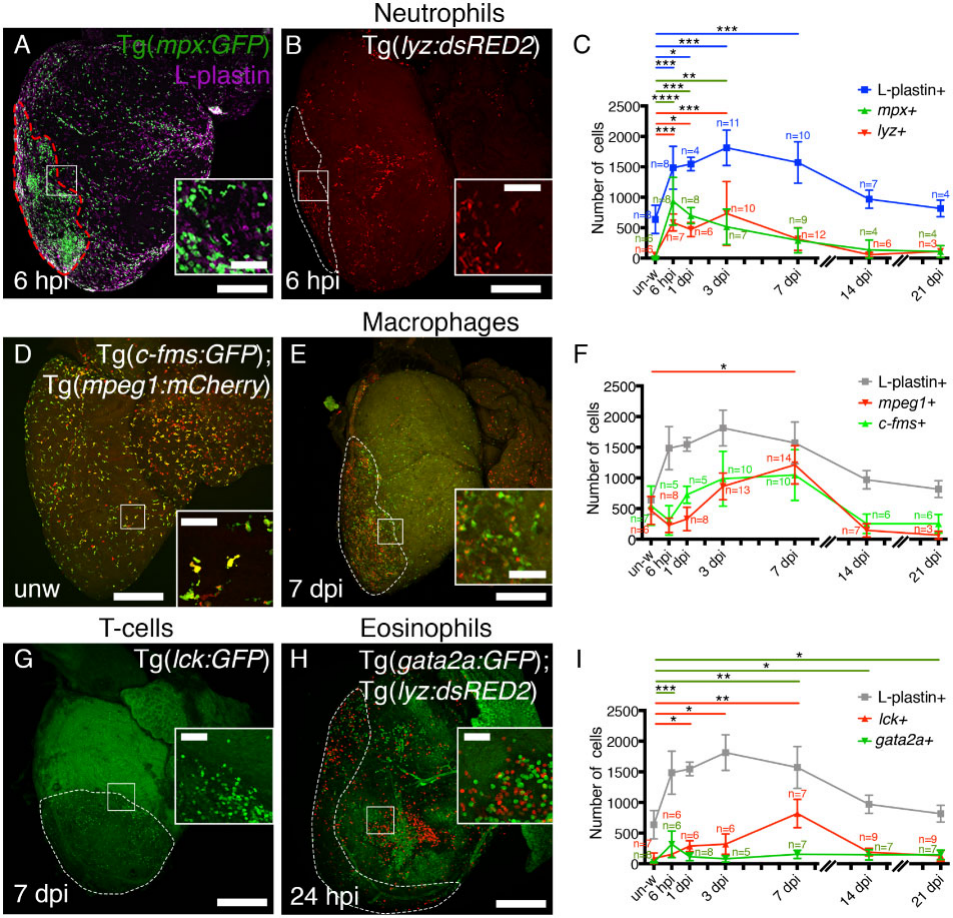
Individual immune cell lineages are predominant at different times post-injury. Representative images of whole hearts dissected from Tg(*mpx:GFP*) (*A*), Tg(*lyz:dsRED2*) (*B*), Tg(*c-fms:GFP*); Tg(*mpeg1:mCherry*) (*D* and *E*), Tg(*lck:GFP*) (*G*), and Tg(*gata2a:GFP*); Tg(*lyz:dsRED2*) (*H*) transgenic zebrafish at the time-points post-injury as indicated. The types of inflammatory cells labelled by the different transgenics are shown above the images. Quantification of the different inflammatory cells across a timeline from unwounded to 21 dpi (*C, F,* and *I*). In *C* the total L-plastin+ cells present at each timepoint is shown in blue. L-plastin+ data also shown in *F* and *I* to allow comparison to total inflammatory cell numbers (same data, grey in *F* and *I*). (*A–C*) Neutrophils [marked by Tg(*mpx:GFP*) (*A*) and Tg(*lyz:dsRED2*) (*B*)] peak at 6 hpi and resolve by 7 dpi (*C*). L-plastin labelling for all leucocytes (in magenta) is also shown in *A*. (*D–F*) Macrophages, marked by Tg(*c-fms:GFP*) and Tg(*mpeg1:mCherry*), are present in large numbers at all time-points and show significant infiltration compared to unwounded hearts at 7 dpi (*F*). The majority of macrophages express both transgenic reporters (inset in *D*). (*G*) Analysis of Tg(*lck:GFP*) fish reveals significant numbers of T-cells at 7 dpi.^33^ Eosinophils, labelled with Tg(*gata2a:GFP*) (*H*), are rarely observed in unwounded hearts but are significantly recruited at 6 hpi (*I*) and at later time-points (7, 14, and 21 dpi) (*I*). Tg(*gata2a:GFP*); Tg(*lyz:dsRED2*) double transgenic fish demonstrate two distinct populations of cells present at 24 hpi (inset in *H*). Boxed regions demark the approximate position of the insets for each panel. Dashed lines in *A, B,* and *E–H* demark the injured region. In *C, F,* and *I*, colour coded *n* numbers are provided for each time-point and transgenic reporter. L-plastin *n* numbers are only shown in *C* for clarity. For statistical analyses Kruskal–Wallis/Dunn’s multiple comparisons tests were used to analyse all data against unwounded for each data set. All other values were not significant. Scale bars: *A, B, D, E, G, H* = 250 μm; insets = 50 μm.

Previous reports have highlighted the importance of different innate and adaptive inflammatory cell populations following cardiac injury and have suggested that some may be required for cardiac regeneration in different species.^11–14^^,^^17^^,^^19^^,^^24^ We next sought to determine the predominant inflammatory cell types that are present at different time-points post-injury in adult zebrafish cardiac tissue. Various transgenic zebrafish lines expressing fluorescently labelled immune cell lineages were cryoinjured and hearts imaged at 6 h post-injury (hpi), 1, 3, 7, 14, and 21 dpi (*Figure 2*). Neutrophils are generally considered to be the first responders to injury^9^ and analysis of either Tg(*mpx:GFP*)i114 or Tg(*lyz:DsRed2*) transgenic zebrafish reveals rapid recruitment of neutrophils following cardiac cryoinjury, peaking at 6 hpi, but with almost complete resolution by 7 dpi (*Figure 2A* and *C*). Although the vast majority of L-plastin+ cells at 6 hpi are neutrophils (*Figure 2A*; Supplementary material online, *Figure S2A*), analysis of Tg(*gata2a:eGFP*) transgenic fish reveals a significant recruitment of eosinophils to early zebrafish cardiac injury also (*Figure 2H* and *I*). Analysis of double transgenic Tg(*gata2a:eGFP*); Tg(*lyz:DsRed2*) fish demonstrates two separate cell populations confirming the specificity of the Tg(*gata2a:eGFP*) fish for eosinophils (*Figure 2H*).

Analysis of two macrophage marker lines, Tg(*mpeg1:mCherry*) and Tg(*c-fms:GFP*) (*csf1ra*), revealed high numbers of macrophages present at all time-points (*Figure 2D–F*). In unwounded hearts, analysis of L-plastin+, *mpeg1+*, and *c-fms+* cell numbers suggests that almost all tissue resident, L-plastin+ cells are macrophages (*Figure 2D* and *F*; Supplementary material online, *Figure S2B*). Significantly elevated numbers of macrophages were present at 7 dpi corresponding to the commencement of resolution (*Figures 1K* and *2E* and *F*). This pattern is very similar to what has been reported for neonatal mice.^13^ Analysis of images in this way provides spatial information on inflammatory cell populations within the heart as well as enabling representative numbers of cells to be quantified. However, to determine the total numbers of macrophages present within the heart, ventricles of Tg(*mpeg1:mCherry*) fish were analysed by FACS at different time-points post-injury (Supplementary material online, *Figure S2C and D*) revealing an average of 0.5 ± 0.1% [unwounded (unw)], 1.2 ± 0.3% (1 dpi), and 7 ± 5% (3 dpi) *mpeg1+* cells per fish (Supplementary material online, *Figure S2C and D*).

Recent reports have suggested a potential role for T-cells in the response to tissue injury^34^^,^^35^ so we investigated the number of T-cells responding to cryoinjury in adult zebrafish using a Tg(*lck:GFP*) transgenic reporter. Significant numbers of *lck+* cells were recruited to the damaged ventricle, peaking at 7 dpi (*Figure 2G* and *I*) and were almost completely resolved by 14 dpi (*Figure 2I*). Additionally, significant numbers of eosinophils were also observed at 7, 14, and 21 dpi (*Figure 2I*). Interestingly, cumulative numbers of *mpx+* (neutrophils), *gata2a+* (eosinophils), *mpeg1+* (macrophages), and *lck+* (T-cells) present at each time-point does not notably differ from the total numbers of L-plastin+ cells suggesting that the cell types labelled by these transgenic markers represent the majority of all inflammatory cells responding to cardiac injury in adult zebrafish (Supplementary material online, *Figure S2E*). Collectively, this analysis demonstrates that multiple different inflammatory cell types respond to cardiac injury in adult zebrafish and are present at similar time points and in similar ratios as observed in mammals. The main difference to adult mammals is the rapid attenuation of the inflammatory response between 7 and 14 dpi.

### 3.3 Macrophage ablation at different time-points significantly alters scar deposition or resolution

**Figure 3:**
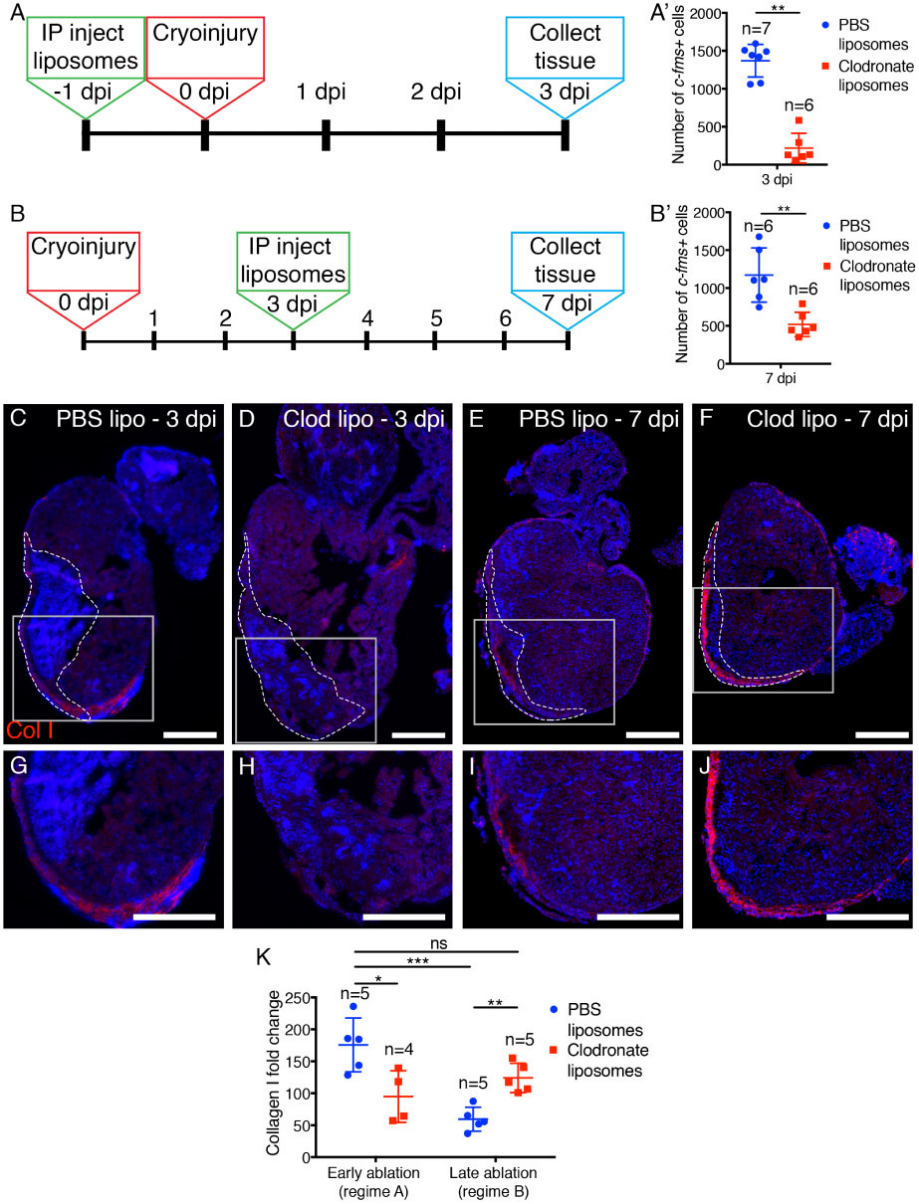
Macrophage ablation results in altered levels of Collagen I. (*A* and *B*) Schematics describing the liposome treatment procedure for early (*A*) and late (*B*) ablation of macrophages. (*A*′ and *B*′) Quantification of the number of *c-fms+* macrophages following either early (regime A; *A* and *A*′) or late (regime B; *B* and *B*′) ablation with clodronate liposomes as compared to PBS liposomes. (*C–J*) Immunofluorescence analysis of Collagen I and subsequent quantification of the fold change compared to unwounded hearts (regime *A, D, H,* and *K*). The quantity of Collagen I is increased in relation to PBS liposome injected following late ablation from 3 to 7 dpi (regime *B, F, J* and *K*). There is no significant difference between control Collagen I levels at 3 dpi and clodronate treated at 7 dpi suggesting a lack of resolution (*K*). The boxed regions in *C–F* demark the approximate position of *G–J*. Dashed lines in *C–F* demark the injured region. For statistical analyses in *A*′ and *B*′, Mann–Whitney tests were used. For statistical analyses in *K*, Mann–Whitney tests were used to analyse PBS vs. Clodronate liposome data in each regime and a Kruskal–Wallis/Dunn’s multiple comparisons test was used to analyse regime A against regime B. Scale bars: *C–J* = 250 μm.

As macrophages are the most abundant inflammatory cell type present in the heart at the crucial scar deposition and Collagen I regression time-points we sought to determine the role(s) of this population. To assess the early function of macrophages (up to 3 dpi), Tg(*c-fms:GFP*) zebrafish received IP injections of clodronate or PBS containing liposomes^36^ on the day prior to cryoinjury with cardiac tissue collected at 3 dpi [*Figure 3A* (regime A)]. To assess the later function of macrophages (3–7 dpi), fish were first cryoinjured and then allowed to recover until 3 dpi when the peak inflammatory and scarring responses are occurring, and only then injected with clodronate or PBS liposomes, and here the repairing cardiac tissue collected at 7 dpi [*Figure 3B* (regime B)]. In both cases the number of *c-fms+* cells was significantly reduced when compared to controls at 3 days post-treatment (*Figure 3A*′ *and B*′ and Supplementary material online, *Figure S3*).

Reducing the number of phagocytic cells during the early injury responses (*Figure 3A*) reduced Collagen I deposition within the myocardium suggesting a role for macrophages in scar formation in line with previous reports (*Figure 3D, H*, and *K*^8^^,^^15^). Conversely, depletion during the later stages of the injury response (*Figure 3B*), resulted in significantly more Collagen I at 7 dpi compared to control fish but no significant difference to control fish at 3 dpi, suggesting a failure in Collagen I resolution (*Figure 3F, J*, and *K*). These observations support previous reports from mammals that macrophages play a role in scar deposition.^8^^,^^15^ In addition, removal of phagocytic macrophages after Collagen I deposition results in a failure of scar resolution suggesting that macrophages are also pivotal in this more novel aspect of the healing process.

### 3.4 A more pro-inflammatory environment affects scarring

**Figure 4:**
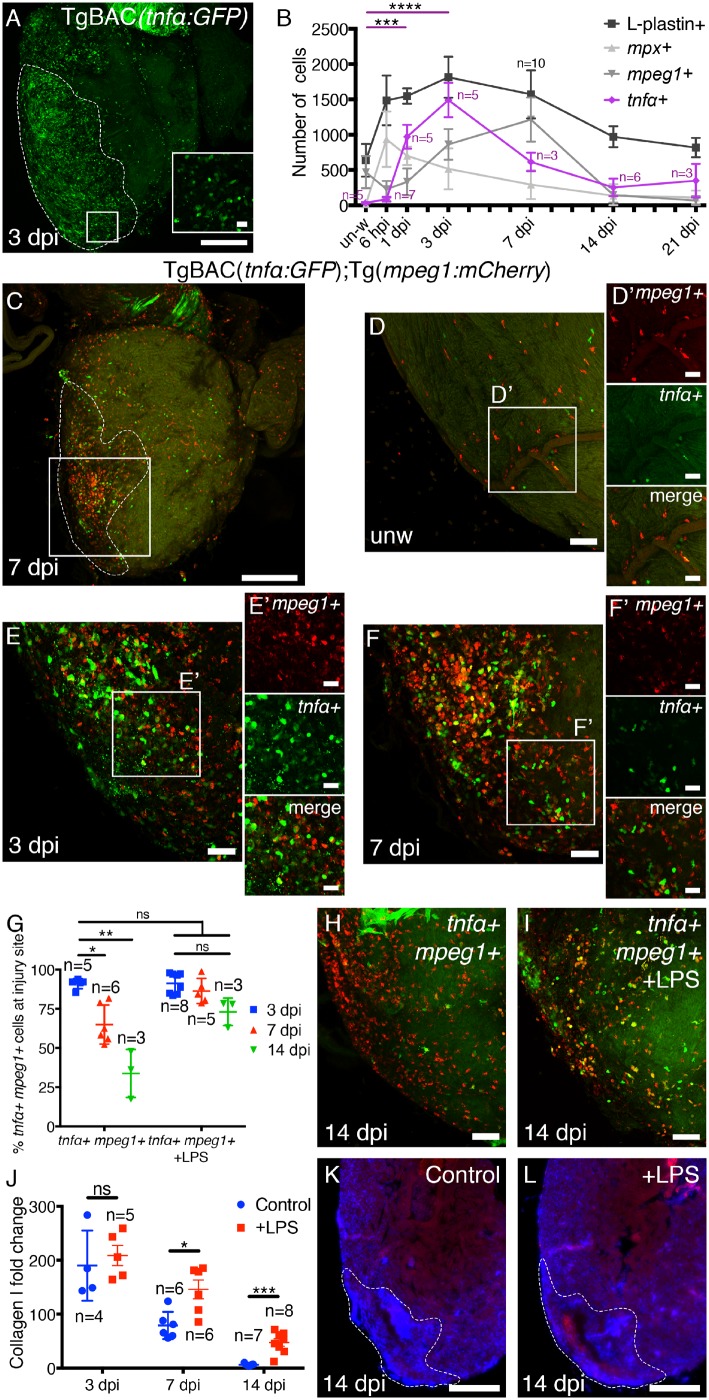
*tnfα+* macrophages respond to cardiac injury and LPS treatment affects Collagen I scarring and results in changes in macrophage phenotype. (*A* and *B*) Representative images and quantification of whole hearts from TgBAC(*tnfα:GFP*) fish at different time-points post-injury. The dashed line in *A* demarks the extent of the injured region. L-plastin, *mpx*, and *mpeg1* data from *Figure 2C* and *F* (greys) are provided as a reference (*B*). (*C*) Representative image of a heart from a Tg(*mpeg1:mCherry*); TgBAC(*tnfα*:*GFP*) double transgenic fish at 7 dpi (higher magnification shown in *F*). The boxed region demarks the approximate area imaged in *D–F* and all subsequent ventricular apex images. (*D–F*) Representative images of the ventricular apex of unwounded Tg(*mpeg1:mCherry*); TgBAC(*tnfα*: *GFP*) fish (*D*) and the injury area at 3 (*E*) and 7 dpi (*F*). Very few *tnfα+ mpeg1+* cells are observed in unwounded hearts (*D*). (*D*′*–F*′) For each image, single channel panels and the merge of the boxed region is provided. (*G*) Quantification of the percentage of *mpeg1+* macrophages at the injury site expressing *tnfα* at 3, 7, and 14 dpi with and without LPS treatment. (*H* and *I*) Representative images of the injury site of Tg(*mpeg1:mCherry*); TgBAC(*tnfα:GFP*) fish (*H*) and following LPS treatment (*I*) at 14 dpi. (*J*) Quantification of the Collagen I fold change as compared to unwounded fish in control and LPS treated fish. (*K–L*) Representative images of sections through the injury site of control (*K*) and LPS treated fish at 14 dpi (*L*). Quantification in *B* and *G*; statistical analysis by Kruskal–Wallis/Dunn’s multiple comparisons tests. *n* numbers for *mpx+*, *mpeg1+*, and L-plastin+ are shown in *Figure 2*. Quantification in *J*; statistical analysis by Mann–Whitney tests at each time-point. un-w, unwounded. Scale bars: *A, C, K, L* = 250 μm; *D–F, H, I* = 50 μm; inset in *A, D*′*–F*′ = 20 μm.

Macrophages exist as a spectrum of different activated phenotypes ranging from pro-inflammatory through to anti-inflammatory/pro-resolution and these subpopulations can, at least partially, be defined by the expression of cytokines and other markers.^20–24^^,^^37^ Next, we used a transgenic reporter line, TgBAC(*tnfα:GFP*), to assess the activation state of the macrophages responding to cardiac injury (*Figure 4*). In unwounded fish, very few *tnfα+* cells were observed whereas there was rapid accumulation of *tnfα*+ cells following cryoinjury with highly significant numbers observed at 1 and 3 dpi (*Figure 4A* and *B*). The number of *tnfα+* cells peaked with the early wave of macrophage influx at 3 dpi but reduced by 7 dpi when peak total macrophage numbers are observed and when scar resolution is commencing (*Figures 1K*, *2F* and *4B*).

To further evaluate the potential opposing roles of these different macrophage populations, we analysed Tg(*mpeg1:mCherry*); TgBAC(*tnfα:GFP*) double transgenic fish in more detail (*Figure 4C–H*). In unwounded fish, very few *tnfα+* macrophages were observed (*Figure 4D* and *D*′). Following cardiac cryoinjury, 91 ± 2% of *mpeg1+* cells were also *tnfα+* at 3 dpi (*Figure 4E, E*′, and *G*). At 7 and 14 dpi, however, this number was significantly reduced (65 ± 5% and 34 ± 9%, respectively; *Figure 4F–H*). We next sought to determine if a more pro-inflammatory environment might influence the scarring response. Treating Tg(*mpeg1:mCherry*); TgBAC(*tnfα:GFP*) fish with a single dose of LPS at the time of injury resulted in significantly more *mpeg1+* macrophages expressing *tnfα* at 7 and 14 dpi when compared to untreated fish (*Figure 4G–I*), although the total number of macrophages was not significantly altered (Supplementary material online, *Figure S4*). Use of LPS to elicit a pro-inflammatory response has recently been validated in zebrafish.^38^ Analysis of the scarring response following these manipulations reveals significantly more Collagen I in the repairing ventricles of fish treated with LPS compared to controls at 7 and 14 dpi, suggesting an association between *tnfα+* macrophages and scar deposition and that limiting the *tnfα+* response is required for scar resolution to occur correctly. With these experiments we cannot completely rule out effects of other cell types (*Figure 4J–L*).

### 3.5 Csf1ra controls macrophage phenotype and drives scarring

**Figure 5:**
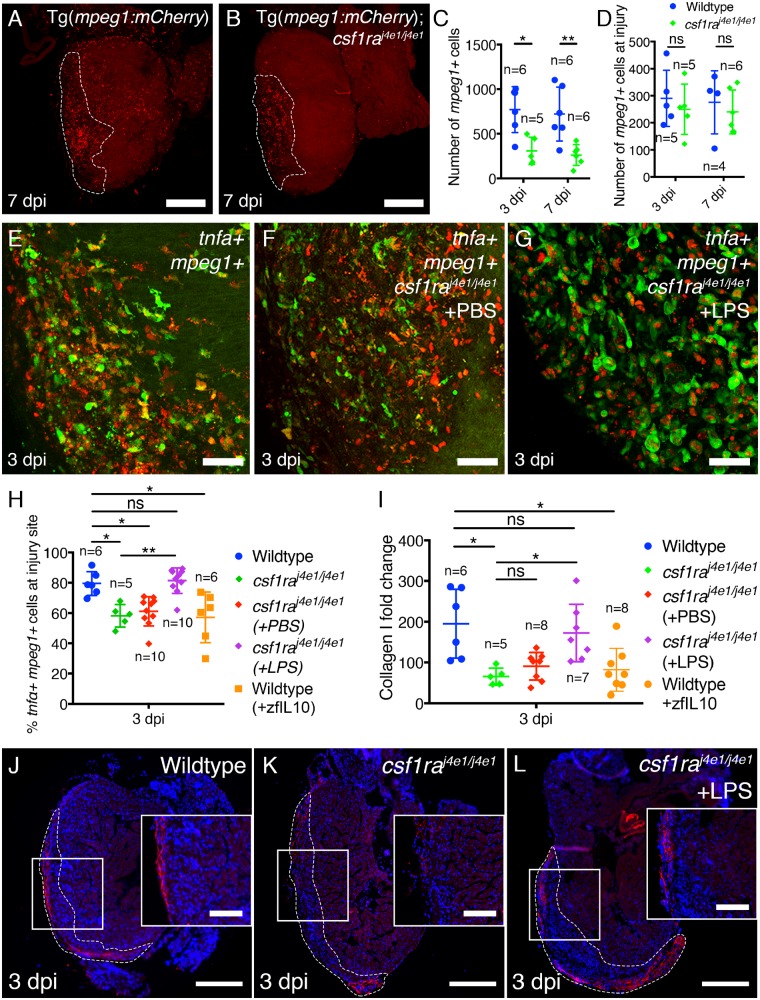
*csf1ra* is required for correct recruitment and a pro-inflammatory phenotype in cardiac macrophages. (*A–D*) Representative images of wildtype Tg(*mpeg1:mCherry*) (*A*) and Tg(*mpeg1:mCherry*); *csf1ra^j^*^4^^*e1/j*^^4^^*e*^^1^ fish (*B*) at 7 dpi and quantification of the total number of macrophages in the ventricle (*C*) and macrophages present at the injury site (*D*). (*E–G*) Representative images of the injury site of TgBAC(*tnfα:GFP*); Tg(*mpeg1:mCherry*) control fish (*E*), TgBAC(*tnfα:GFP*); Tg(*mpeg1:mCherry*); *csf1ra^j^*^4^^*e1/j*^^4^^*e*^^1^ treated with PBS (*F*), and TgBAC(*tnfα:GFP*); Tg(*mpeg1:mCherry*); *csf1ra^j^*^4^^*e1/j*^^4^^*e*^^1^ treated with LPS (*G*) at 3 dpi. (*H*) Quantification of the percentage of *mpeg1+* macrophages at the injury site expressing *tnfα* in control, TgBAC(*tnfα:GFP*); Tg(*mpeg1:mCherry*); *csf1ra^j^*^4^^*e1/j*^^4^^*e*^^1^, TgBAC(*tnfα:GFP*); Tg(*mpeg1:mCherry*); *csf1ra^j^*^4^^*e1/j*^^4^^*e*^^1^ fish treated with PBS, TgBAC(*tnfα:GFP*); Tg(*mpeg1:mCherry*); *csf1ra^j^*^4^^*e1/j*^^4^^*e*^^1^ fish treated with LPS and wildtype TgBAC(*tnfα:GFP*); Tg(*mpeg1:mCherry*) fish treated with recombinant zebrafish IL10. (*I*) Quantification of the Collagen I fold-change in wildtype, *csf1ra^j^*^4^^*e1/j*^^4^^*e*^^1^ fish, *csf1ra^j^*^4^^*e1/j*^^4^^*e*^^1^ fish treated with PBS, *csf1ra^j^*^4^^*e1/j*^^4^^*e*^^1^ fish treated with LPS, and wildtype fish treated with recombinant zebrafish IL10 at 3 dpi. (*J–L*) Representative images of Collagen I staining in wildtype (*J*), *csf1ra^j^*^4^^*e1/j*^^4^^*e*^^1^ (*K*), and *csf1ra^j^*^4^^*e1/j*^^4^^*e*^^1^ fish treated with LPS (*L*). Dashed lines in *A, B*, and *J–L* demark the injured region. Boxed regions in *J–L* demark the approximate position of the inset. Quantification in *C* and *D*; statistical analysis by Mann–Whitney tests of control and *csf1ra^j^*^4^^*e1/j*^^4^^*e*^^1^ data at each time-point. Quantification in *H* and *I*; statistical analysis by Kruskal–Wallis/Dunn’s multiple comparisons tests. Scale bars: *A, B, J–L* = 250 μm; *E–G* = 100 μm; inset in *J–L* = 100 μm.

We next assessed the inflammatory and scarring response to cardiac injury in *csf1ra^j^*^4^^*e1/j*^^4^^*e*^^1^ mutant zebrafish. Csf1ra has been suggested to be important for macrophage differentiation and polarization and null Csf1r mouse mutants have reduced numbers of macrophages.^39^ As expected, analysis of *csf1ra^j^*^4^^*e1/j*^^4^^*e*^^1^; Tg(*mpeg1:mCherry*) zebrafish revealed significantly reduced numbers of *mpeg1+* macrophages at 3 and 7 dpi across the whole ventricle (*Figure 5A–C*). Numbers of *mpeg1+* macrophages present only at the injury site in *csf1ra^j^*^4^^*e1/j*^^4^^*e*^^1^; Tg(*mpeg1:mCherry*) fish, however, were similar to control fish suggesting that the remaining macrophages are functional and responsive to injury (*Figure 5B* and *D*). Interestingly, analysis of *csf1ra^j^*^4^^*e1/j*^^4^^*e*^^1^; Tg(*mpeg1:mCherry*); TgBAC(*tnfα:GFP*) fish revealed a significant reduction in the number of *tnfα+* macrophages at the injury site at 3 dpi compared to wildtype (58 ± 3%; *Figure 5E*, *F* and *H*) and these fish exhibited significantly reduced collagen deposition (*Figure 5I–K*). LPS treatment of *csf1ra^j^*^4^^*e1/j*^^4^^*e*^^1^; Tg(*mpeg1:mCherry*); TgBAC(*tnfα:GFP*) fish partially rescued the number of *tnfα+* macrophages responding to cardiac injury (81 ± 3%; *Figure 4G* and *H*) and this rescue was associated with significantly increased Collagen I deposition at 3 dpi suggesting a rescue of the scarring response also (*Figure 5I* and *L*).

To further address whether an initial macrophage pro-inflammatory response is required for scar deposition we treated wildtype Tg(*mpeg1:mCherry*); TgBAC(*tnfα:GFP*) fish with recombinant zfIL10, a cytokine that promotes anti-inflammatory polarization in macrophages, at the time of injury (*Figure 5H* and *I* and Supplementary material online, *Figure S5A–D*). This treatment reduced the number of *tnfα+* macrophages at the injury at 3 dpi (58 ± 7%; *Figure 5H* and Supplementary material online, *Figure S5A and B*) and the amount of Collagen I, recapitulating the *csf1ra^j^*^4^^*e1/j*^^4^^*e*^^1^ fish (*Figure 5I* and Supplementary material online, *Figure S5C and D*). We also determined the number of proliferating cells present in the ventricle of wildtype and *csf1ra^j^*^4^^*e1/j*^^4^^*e*^^1^ fish at 7 dpi. No significant differences were observed (Supplementary material online, *Figure S5E–G*). Collectively, our data suggest that an early pro-inflammatory response in macrophages, which requires Csf1ra, is necessary to initiate the scarring response in adult zebrafish but loss of *csf1ra* does not affect proliferation. Also, between 3 and 7 dpi, macrophages reduce *tnfα* expression and inhibiting this switch in phenotype with LPS treatment blocks scar resolution.

### 3.6 Opn is expressed in a subset of macrophages during scar resolution

**Figure 6:**
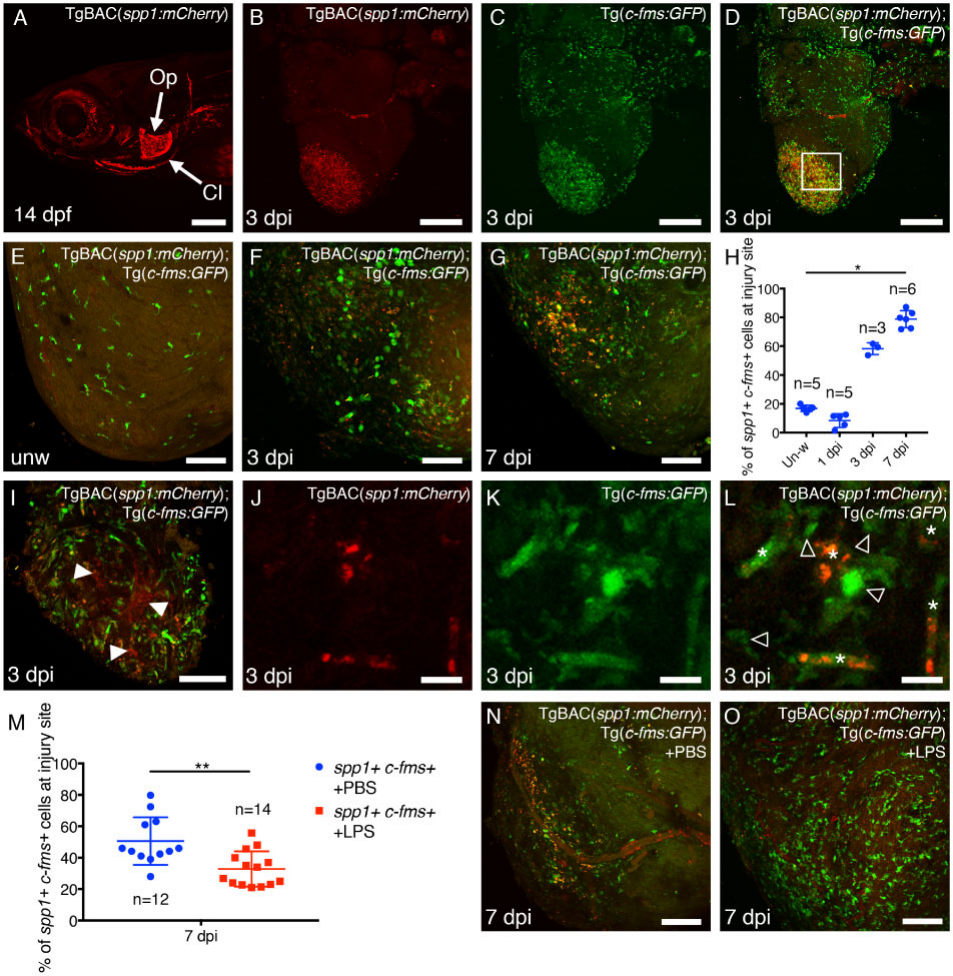
TgBAC(*spp1:mCherry*) fish reveal *spp1* expression in a subset of macrophages following cardiac injury. (*A*) Lateral image of the head of a TgBAC(*spp1:mCherry*) fish at 14 dpi demonstrating *spp1* expression in the craniofacial skeleton, as reported previously.^44^ Anterior is to the left. Op, Operculum, Cl, Cleithrum. (*B–D*) Images of an adult heart from a TgBAC(*spp1:mCherry*); Tg(*c-fms:GFP*) fish demonstrating *spp1* expression specifically at the injury site at 3 dpi (*B*), partially co-localizing with *c-fms* expression (*C* and *D*). (*E–G*) Confocal images of the apex of the ventricle of unwounded (*E*), 3 dpi (*F*), and 7 dpi (*G*) hearts from TgBAC(*spp1:mCherry*); Tg(*c-fms:GFP*) double transgenic fish. Very little *spp1* expression is seen in macrophages of unwounded fish (*E*). (*H*) Quantification of the percentage of *c-fms+* macrophages at the injury site that are expressing *spp1*. (*I*) Single *z*-plane of the boxed region in *D*. Other cells in the injury site also express *spp1* (arrowheads). (*J–L*) *spp1* is expressed in a subset of macrophages in the injured heart at 3 dpi (asterisks in *L*). Open arrowheads indicate *c-fms+* macrophages not expressing *spp1*. (*M–O*) Quantification and representative images of the injury site of TgBAC(*spp1:mCherry*); Tg(*c-fms:GFP*) double transgenic fish following treatment with PBS (Control; *N*) or LPS (*O*). Statistical analysis in *H*; Kruskal–Wallis/Dunn’s multiple comparisons test. In *M*, statistical analysis was performed by a Mann–Whitney test. Scale bars: *A* = 200 μm, *B–D* = 250 μm, *E–G, I, N, O* = 100 μm, *J–L* = 10 μm.

One gene shown to be downstream of the injury inflammatory response and causal of fibrosis in mouse is *spp1*, encoding Opn.^26^^,^^27^^,^^40^ Opn is a multifunctional protein that plays intra- and extracellular roles both as a transcription factor and as a secreted factor that can bind integrins.^25^^,^^41–43^ Opn is expressed by both macrophages and fibroblasts following tissue injury in mammals^26^ and *Spp1* has been shown to be up-regulated in pro-resolving macrophages in the mouse heart;^24^ however, Opn function has not been studied during tissue regeneration. To determine the cell types expressing *spp1* in response to cardiac cryoinjury, we generated a reporter line—TgBAC(*spp1:mCherry*) (*Figure 6*). Analysis of this reporter line reveals that mCherry expression recapitulates the previously described expression pattern of *spp1* during development (*Figure 6A*; Supplementary material online, *Figure S6A–C*).^44^ Analysis of hearts from adult TgBAC(*spp1:mCherry*); Tg(*c-fms:GFP*) fish reveals *spp1* expression specifically at the site of cardiac injury at 3 dpi, partly co-localizing with Tg(*c-fms:GFP*) (*Figure 6B–D*). *In situ* hybridization confirms the specificity of the TgBAC(*spp1:mCherry*) line in the craniofacial skeleton and the heart (Supplementary material online, *Figure S6A–F*).

Detailed analysis of TgBAC(*spp1:mCherry*); Tg(*c-fms:GFP*) fish demonstrates a subset of *spp1+ c-fms+* macrophages following cardiac injury (*Figure 6E–L*) with a significant increase at 7 dpi (*Figure 6E–H*). Very few *spp1+ c-fms+* cells are observed in unwounded hearts or at 1 dpi (*Figure 6E* and *H*). At 3 dpi, another subset of *spp1+* cells are *c-fms*− and are most likely interstitial fibroblasts (*Figure 6I*^26^). Indeed, analysis of TgBAC(*spp1:mCherry*); *ET37* fish suggests localization of *spp1* in a subset of cardiac fibroblasts (Supplementary material online, *Figure S6G*). The enhancer trap line, *ET37*, has been shown to label larval mesenchymal cells and we have further verified this line as labelling cardiac fibroblasts (Supplementary material online, *Figure S7*^45^). Analysis of TgBAC(*spp1:mCherry*); TgBAC(*tnfα:GFP*) fish at 3 and 7 dpi reveals double *spp1+*; *tnfα+* and single positive cells suggesting dynamic expression changes of these two macrophage phenotype markers (Supplementary material online, *Figure S6H and I*). To further identify the macrophages expressing *spp1* following cardiac injury we treated TgBAC(*spp1:mCherry*); Tg(*c-fms:GFP*) fish with LPS at the time of injury (*Figure 6M–O*) which significantly reduced the number of *spp1*+ macrophages suggesting these are more anti-inflammatory cells. Together, our data suggest that TgBAC(*spp1:mCherry*) fish recapitulate the developmental expression pattern of *spp1*, demonstrate expression in a subset of cell types in the heart, specifically after injury and that this expression is reduced with LPS treatment.

### 3.7 Opn is required for macrophage phenotypic switching, inflammatory response resolution, and scar remodelling

**Figure 7:**
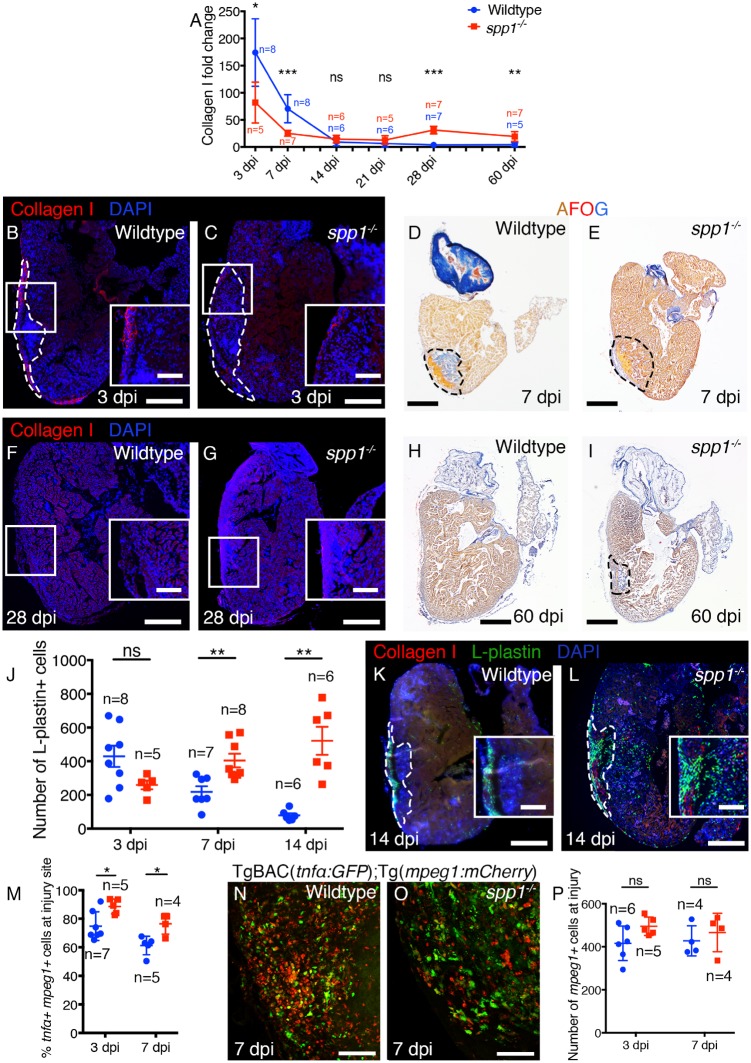
Loss of *spp1* results in an extended inflammatory response and altered scarring. (*A*) Quantification of Collagen I in the ventricle of wildtype vs. *spp1*^−/−^ mutants at the time-points indicated. (*B–I*) Representative images of immunofluorescence analysis for Collagen I (*B, C, F,* and *G*) and AFOG (collagen in blue; *D, E, H,* and *I*) on sections through the ventricle of wildtype (*B, D, F,* and *H*) and *spp1*^−/−^ mutants (*C, E, G,* and *I*) at 3, 7, 28, and 60 dpi. (*J*) Quantification of the number of L-plastin+ cells present in the ventricle of wildtype and *spp1*^−/−^ mutants at the time-points indicated. (*K* and *L*) Representative images of immunofluorescence analysis of L-plastin and Collagen I on sections through the ventricle of wildtype (*K*) and *spp1*^−/−^ mutants (*L*) at 14 dpi. (*M*) Quantification of the percentage of *mpeg1+* macrophages at the injury site expressing *tnfα* in wildtype TgBAC(*tnfα:GFP*); Tg(*mpeg1:mCherry*) and *spp1*^−/−^; TgBAC(*tnfα:GFP*); Tg(*mpeg1:mCherry*) fish at 3 and 7 dpi. (*N* and *O*) Representative images of the injury site of wildtype TgBAC(*tnfα:GFP*); Tg(*mpeg1:mCherry*) (*N*) and *spp1*^−/−^; TgBAC(*tnfα:GFP*); Tg(*mpeg1:mCherry*) fish at 7 dpi (*O*). (*P*) Quantification of the number of *mpeg1+* macrophages at the injury site in wildtype and *spp1*^−/−^ mutants at 3 and 7 dpi. Dashed lines in *B–E, I, K,* and *L* demark the injured region. Boxed regions in *B, C, F,* and *G* demark the approximate position of the inset. Statistical analysis in *A, J, M,* and *P* by Mann–Whitney tests between wildtype and mutant at each time-point. Scale bars: *B–I, K, L* = 250 μm; inset in *B, C, F, G, K, L* = 50 μm; *N, O* = 100 μm.

To determine whether zebrafish Opn plays a role in regulating macrophage phenotype during cardiac repair/regeneration, we generated *spp1* mutant lines [*spp1^C331del/C331del^*/*spp1^CGAT327-330del/C327-330del^* (referred to as *spp1*^**−/−**^); *Figure 7* and Supplementary material online, *Figure S8*]. These mutations result in frameshifts and a premature STOP codon that removes the majority of Opn functional domains predicted from mammals (Supplementary material online, *Figure S8A and B*) although we cannot currently confirm a complete lack of protein in these fish. The resulting adult fish appear grossly normal (Supplementary material online, *Figure S8C and D*). Analysis of the scarring phenotype in *spp1*^**−/−**^ fish reveals a significant reduction in Collagen I deposition following cryoinjury at 3 and 7 dpi, similar to that described for Opn inhibition following skin wounding in mouse (*Figure 7A–E*^26^). Interestingly, the amount of scarring in the ventricle was significantly increased at later time points of 28 and 60 dpi by either Collagen I immunofluorescence or AFOG, which labels all collagens (*Figure 7A* and *F–I*), suggesting that zebrafish Opn plays a role in scar resolution also. To assess effects of *spp1* mutation on cardiomyocyte regeneration we assessed proliferation in wildtype and *spp1*^**−/−**^ fish at 7 dpi (Supplementary material online, *Figure S8E–G*). No significant differences were observed.

Quantification of L-plastin+ cells responding to cardiac injury in *spp1*^**−/−**^ fish reveals significantly higher numbers compared to wildtype at 7 and 14 dpi (*Figure 7J–L*) suggesting a failure in the resolution of the inflammatory response in the absence of Opn. Further analysis of *spp1*^**−/−**^; Tg(*mpeg1:mCherry*); TgBAC(*tnfα:GFP*) fish reveals increased numbers of *tnfα+* macrophages at 3 and 7 dpi compared to wildtype fish (*Figure 7M–O*) although normal numbers of total macrophages are observed at the injury site (*Figure 7P*). This suggests that Opn is critical for controlling the switch in macrophage phenotype between 3 and 7 dpi from *tnfα+* to *tnfα*− and that this is required for later resolution of inflammation and may impact on scar remodelling/resolution. Together, these data suggest bifunctional roles for Opn in both fibrosis/scar deposition, as in mammals, but also in regulation of inflammation and promotion of scar resolution, highlighting a previously unidentified role of Opn as a contributor to tissue regeneration.

## 4. Discussion

Cardiac scarring following tissue damage is a major contributing factor to the progression of heart failure, a leading cause of death in the western world. One major therapeutic goal is to improve the capacity of mammalian hearts to undergo regenerative rather than fibrotic repair. Interventions under investigation include generation of induced pluripotent stem cells (iPSCs) or differentiated cardiomyocytes and subsequent implantation into the infarcted region of the heart.^46^^,^^47^ Currently, however, the efficacy of these interventions remains limited.^47^ One factor limiting the success of these therapeutic strategies could be the adverse environment in which these cells are implanted. Scar tissue represents an abnormal, stiffened extracellular architecture that limits the connectivity and function of the cells embedded within it.^48^ Therefore, combined therapeutics that both provide replacement cells/trophic signals and limit the changes to, or improve, the extracellular environment at the site of an infarct could have major benefits.

The inflammatory response to tissue injury is a necessary consequence but one that drives subsequent fibrosis and scar formation.^6^^,^^8^^,^^9^ In this study, we demonstrate that adult zebrafish exhibit similar relative contributions of inflammatory cell types and scarring following cardiac injury to mammals; however, the inflammatory response is much more rapidly attenuated and subsequent scar resolution occurs. Our data indicates that different macrophage populations promote both the formation of a cardiac scar and subsequent scar resolution/regeneration. This supports recent reports demonstrating a requirement for macrophages as a whole for cardiac and limb regeneration.^17–19^ These recent reports described global leucocyte and neutrophil and macrophage infiltration to the heart and used depletion of macrophages with clodronate at the time of injury to demonstrate a requirement for these cells in promoting cardiomyocyte proliferation and in neutrophil clearance.^17^^,^^19^ Our findings complement and extend these previous studies by demonstrating the involvement of multiple inflammatory cell types and highlighting the importance of macrophage phenotype in regenerative processes. By manipulating macrophage subsets, either by global reduction or by influencing activation state, we demonstrate that *tnfα+* macrophages promote scar deposition, but a more anti-inflammatory phenotype is necessary to allow scar resolution. Csf1ra is required to maintain normal numbers of macrophages in the whole heart but the numbers responding to injury are relatively normal in *csf1ra^j^*^4^^*e1/j*^^4^^*e*^^1^ mutants. However, these fish exhibit significantly reduced *tnfα+* macrophages and scarring, supporting a role for these pro-inflammatory cells in promoting scar deposition, although the overall reduced numbers of cardiac macrophages may contribute to the reduced scarring observed in these fish. *In vitro* data suggests that human pro-inflammatory (‘M1’) macrophages are less fibrotic than alternatively activated (‘M2’) macrophage populations;^49^ however, our data suggest that this view may be too simplistic and that more dynamically activated macrophage subpopulations may influence the balance between a pro- and anti-fibrotic milieu and that zebrafish are uniquely placed to decipher these dynamic activation states.

We have also identified a role for zebrafish Opn both in promoting scarring but also as a pro-regenerative factor in the heart. OPN is a multifunctional protein shown to be a pro-fibrotic factor downstream of the inflammatory response in mammals.^26^^,^^27^^,^^40^ OPN regulates mammalian haematopoietic stem cell homing to the bone marrow niche,^50^ a model of cellular regeneration, but its role in tissue regeneration is not known. We demonstrate that Opn is expressed in a subset of macrophages at the injury, with an increase in *spp1+* cardiac macrophages at 7 dpi, a reciprocal expression pattern from that observed for *tnfα*. Studies in mammals have described contradictory roles for OPN in controlling macrophage phenotype; it may be a marker of alternatively activated ‘M2’ macrophages in the mouse heart^24^ but loss of OPN promotes a more anti-inflammatory phenotype in a model of muscular dystrophy.^51^ We have used *tnfα* and *spp1* expression to define macrophage activation state, but further investigation will be required to delineate the precise phenotypes of macrophage populations in the adult zebrafish heart and to determine differences from mammalian macrophages, perhaps further illustrating why scar removal is so much more effective in zebrafish than in mammals.

*spp1* ^**−/−**^ fish exhibit reduced collagen deposition at 3 and 7 dpi but they exhibit significantly more scarring at 28 and 60 dpi when compared to wildtype fish, indicating a failure in resolution. Our data supports a role for Opn in specifically promoting Collagen I deposition, as observed in mammals, as this is the predominant collagen present at early stages post-injury. Interestingly, a recent report suggests collagens other than Collagen I are expressed during zebrafish cardiac fibrosis and scarring, particularly at later stages,^32^ and our data supports this. Further work will be required to determine if Opn effects Collagen I and other collagens differently. We have shown that Opn promotes scarring, but also functions as a regenerative factor by facilitating the rapid attenuation of the inflammatory response, which characterizes the zebrafish response to cardiac injury. *spp1*^**−/−**^ fish exhibit an uncontrolled and expanded inflammatory response at 7 and 14 dpi and a concurrent increase in the number of *tnfα+* macrophages, further supporting the role of Opn in control of the inflammatory response to cardiac injury. Analysis of proliferation rates in either the whole ventricle or specifically in Tropomyosin+ cardiomyocytes did not reveal significant differences between *csf1ra^j^*^4^^*e1/j*^^4^^*e*^^1^, *spp1*^**−/−**^, and wildtype fish suggesting cardiomyocyte regenerative capacity is unaffected by the loss of *csf1ra* or *spp1* and that this regenerative process is uncoupled from scarring and scar resolution. However, *spp1*^**−/−**^ fish fail to completely remove scar tissue demonstrating that modulation of abnormal deposited scarring is a pre-requisite for complete tissue regeneration, regardless of cardiomyocyte proliferative ability.

Collectively, our data suggest that adult zebrafish possess an intricately balanced inflammatory response to cardiac injury, with similarities to that described in mammals. However, zebrafish utilize an initial pro-inflammatory response to drive Collagen I scar deposition rapidly following ventricular damage and we have demonstrated that *csf1ra* is crucial for this early pro-inflammatory response and scarring. Subsequently, zebrafish macrophages exhibit a rapid phenotypic switch to reduce *tnfα* and increase *spp1* expression and that this switch is necessary to allow inflammatory and scar resolution to occur. Our data provide evidence to suggest that the subtle regulation and balance of the inflammatory response and in particular of macrophage phenotypic state is crucial for scar resolution and subsequent full cardiac regeneration.

**Translational perspective**

Currently, curative treatments for severe cardiac scarring/heart failure include complex surgical interventions and, ultimately, a complete heart transplant. Therapeutic interventions that aim to improve the limited intrinsic regenerative potential of mammalian hearts or to reconstitute lost cardiomyocytes via exogenous stem cells or iPSCs are under intense investigation but have shown limited success, potentially due to the adverse scarred microenvironment these cells are placed into.^46^^,^^47^ Combining these therapies with future treatments that promote beneficial modulation of the extracellular microenvironment of the heart could have major benefits for the field of cardiovascular regenerative medicine.

## Supplementary Material

cvz221_Supplementary_Data
