# Supplementary material for: Specific macrophage populations promote both cardiac scar deposition and subsequent resolution in adult zebrafish
Source: Cardiovasc Res. 2019 Aug 16;116(7):1357–71. doi: 10.1093/cvr/cvz221 (PMC7243279; doi:10.1093/cvr/cvz221)
Supplement: cvz221_Supplementary_Data [file cvz221_supplementary_data.docx]

**SUPPLEMENTARY MATERIAL**

**Supplementary Figures**

**
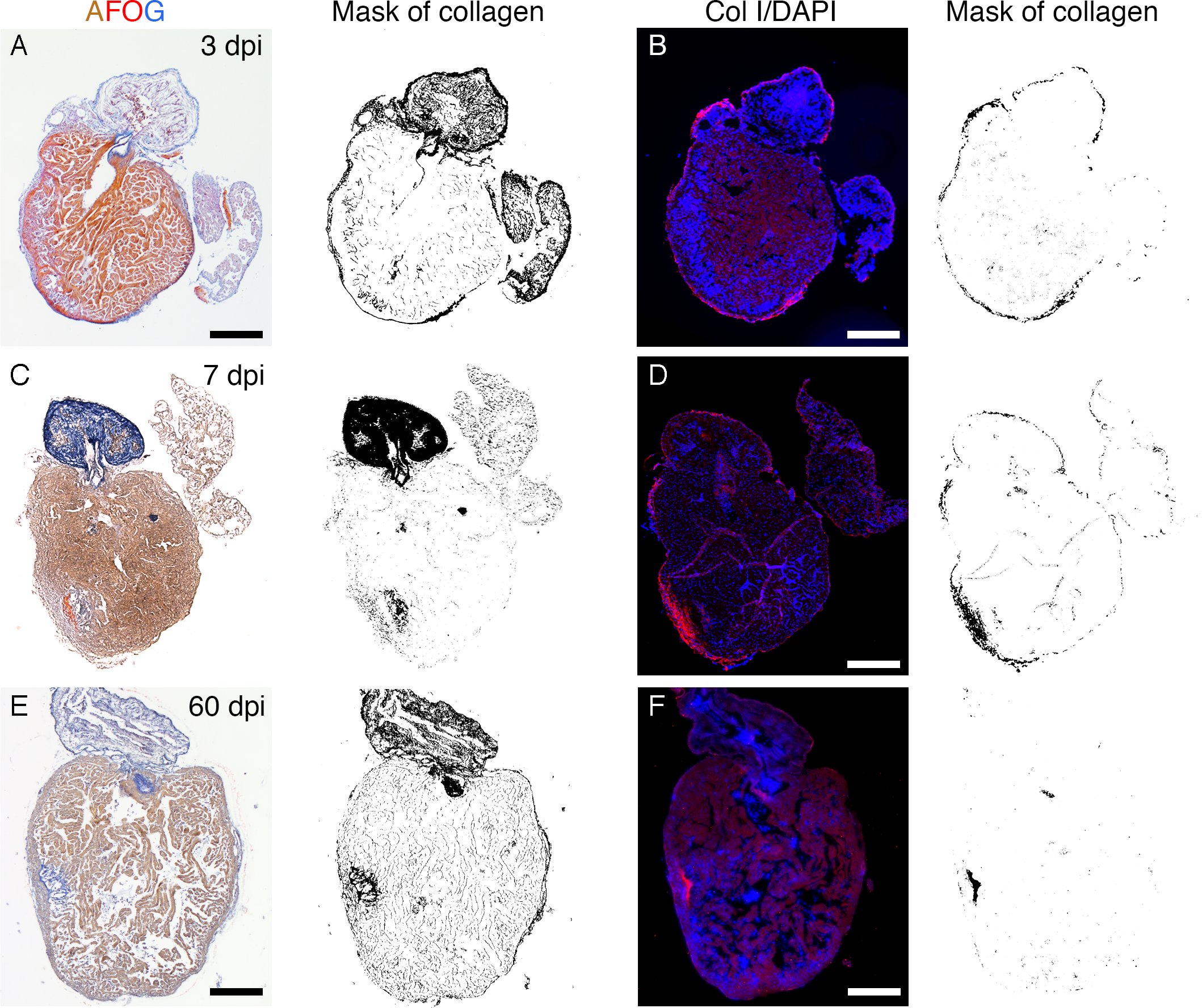
**

**Figure S1 – Comparison of AFOG and Collagen I staining.** (A-F) Comparison of AFOG histological staining (A,C,E) and Collagen I immunostaining (B,D,F) on sections from adjacent slides of the same heart at 3 (A,B), 7 (C,D) and 60 dpi (E,F). For each image the mask obtained from applying a colour threshold in ImageJ is shown alongside the original image to aid in visualisation of the specific staining for collagen. For AFOG this colour threshold was set to filter only blue and for Collagen I the colour threshold was set to filter only red. The same parameters were used for each set of images. Scale bars: 250 μm.

**
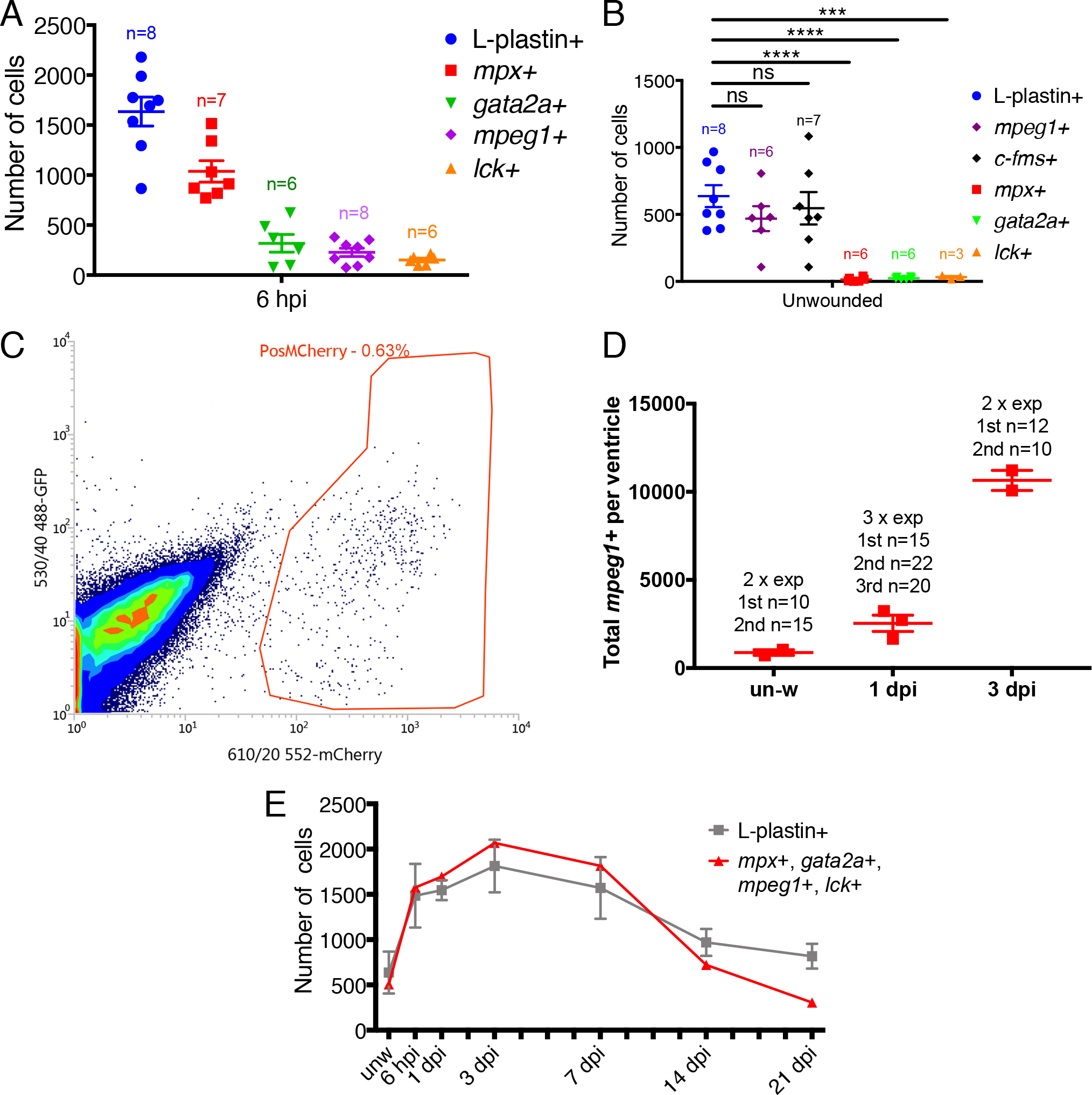
**

**Figure S2 – Neutrophils, eosinophils, macrophages and T-cells make up the majority of inflammatory cell types responding to cardiac injury.** (A) Comparison of the number of L-plastin+, *mpx+,* *gata2a+,* *mpeg1+* and *lck+* present in the ventricle at 6 hpi. The majority of L-plastin+ cells are neutrophils. This is the same data as shown in Figure 2C, F and I. (B) Quantification of the different cell types present in unwounded cardiac tissue. There is no significant difference between the number of L-plastin+ cells and *mpeg1+* or *c-fms+* suggesting that the majority of the cells observed in the unwounded situation are macrophages. This is the same data as shown in Figure 2C, F and I. (C) Representative FACS plot of the percentage of *mCherry+* macrophages sorted from an unwounded adult Tg(*mpeg1:mCherry*) ventricle. (D) Quantification of the total number of *mpeg1+* macrophages present in the ventricle of adult Tg(*mpeg1:mCherry*) fish at the time-points indicated. In each case each point represents a single experiment containing pooled hearts (pooled heart numbers =10-22 per experiment as indicated). The data is presented as the average number of cells per ventricle per experiment. (E) Comparison of the number of L-plastin+ cells (grey) responding to cardiac injury in whole hearts of unwounded (unw), 6 hpi, 1, 3, 7, 14 and 21 dpi adult zebrafish with the sum of the average number of *mpx+,* *gata2a+,* *mpeg1+* and *lck+* (red). n numbers are shown in Figure 2I (L-plastin) or are the sum of n numbers for *mpx+,* *gata2a+,* *mpeg1+* and *lck+* shown in Figure 2C,F,I. For statistical analyses in A,B, Kruskal-Wallis/Dunn’s multiple comparisons tests were used to analyse all data against L-plastin.

**
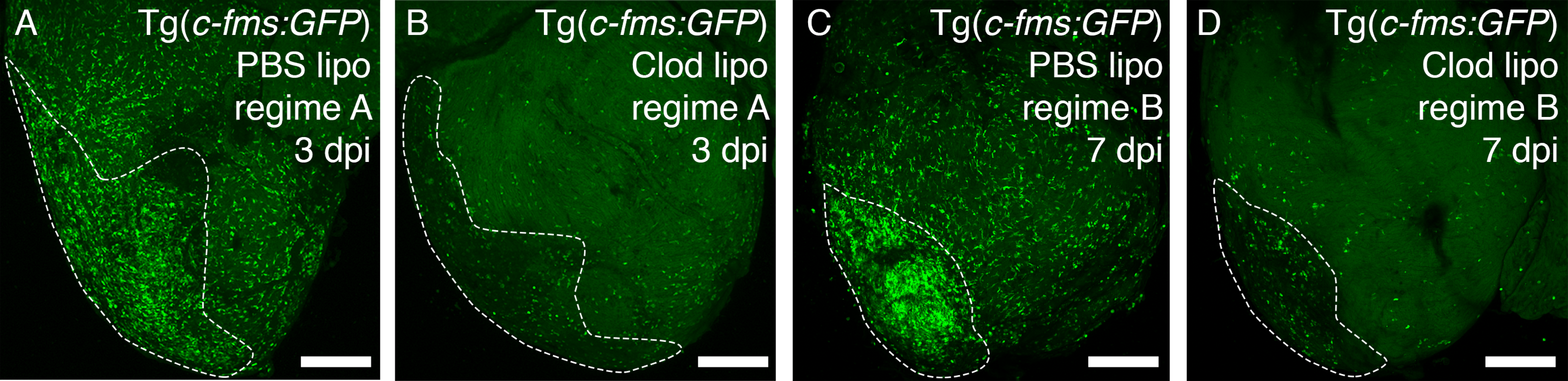
**

**Figure S3 –Clodronate treatment reduces the number of *c-fms+* macrophages responding to cardiac injury.** (A-D) Representative images of Tg(*c-fms:GFP*) fish at 3 dpi (A,B) and 7 dpi (C,D) following IP injection of PBS liposomes one day prior to cryoinjury (A) or at 3 dpi (C). IP injection of clodronate liposomes one day prior to cryoinjury (B) or at 3 dpi (D) reduces the number of macrophages in the ventricle. The dashed lines demark the extent of the injured region. Scale bars: 250 μm.

**Figure S4 – The number of *mpeg1+* cells at the injury does not significantly alter following LPS treatment.** Quantification of the total number of *mpeg1+* cells at the injury site of control Tg(*mpeg1:mCherry*) fish or following treatment with LPS at the time of injury. This is the sum of the *mpeg1+* cells from the same fish presented in Figure 4G. For statistical analysis, Mann-Whitney tests were used to test control and LPS treated at each time-point.

**
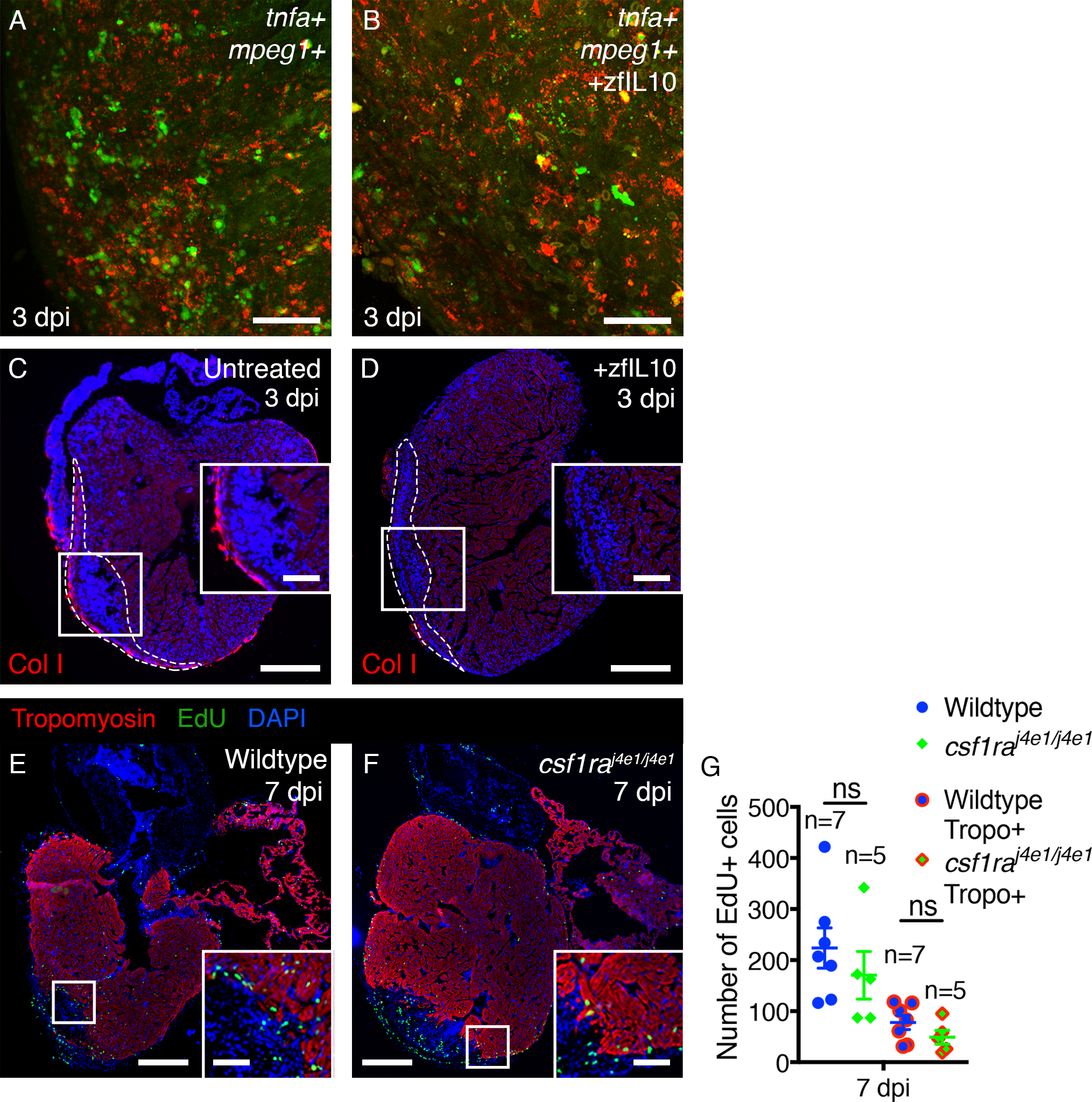
**

**Figure S5 – Recombinant zebrafish IL10 treatment reduces scar deposition and proliferation rates are normal in *csf1ra^j4e1/j4e1^* fish.** (A,B) Representative images of the injury site of a control TgBAC(*tnfα:GFP*); Tg(*mpeg1:mCherry*) fish (A) and a TgBAC(*tnfα:GFP*); Tg(*mpeg1:mCherry*) fish treated with recombinant zebrafish IL10 (zfIL10), at 3 dpi (B). (C,D) Representative images of sections through the heart of a control, untreated fish (C) and a fish treated with zfIL10 (D) following immunostaining of Collagen I, at 3 dpi. The dashed lines in C,D demark the extent of the injured region. The boxed regions in C,D demark the approximate position of the inset. (E-G) Representative images (E,F) and quantification (G) of the number of EdU+, proliferative cells in wildtype (E) and *csf1ra^j4e1/j4e1^* fish (F) at 7 dpi. The number of Tropomyosin+, EdU+ positive cardiomyocytes were also quantified (G). In G, statistical analysis was performed by Mann-Whitney tests of control and *csf1ra^j4e1/j4e1^* data. Scale bars: A,B = 100 μm; C-F = 250 μm; inset in E,F = 50 μm.

**
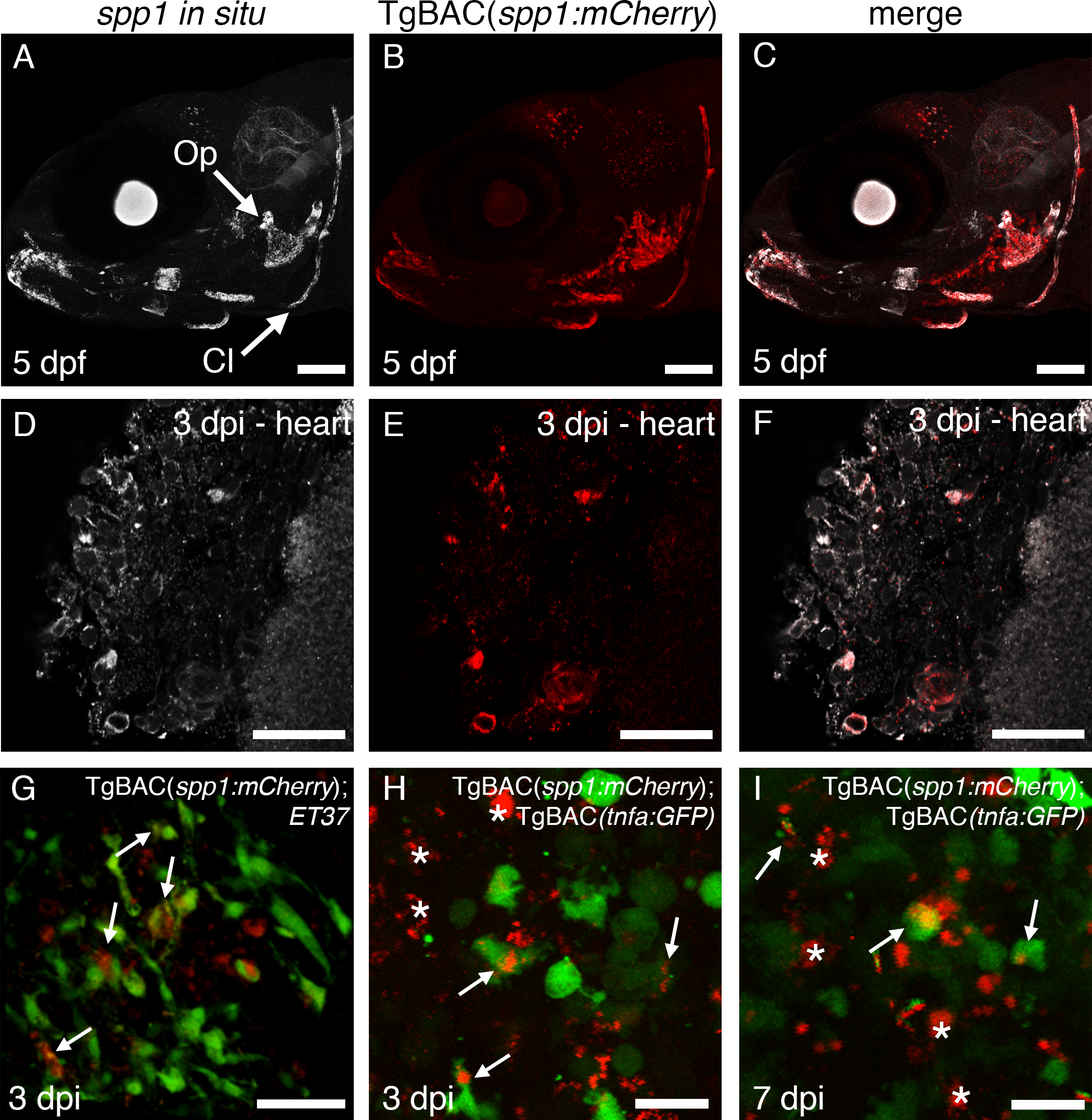
**

**Figure S6 – TgBAC(*spp1:mCherry*) transgenic fish recapitulate the *in situ* hybridisation expression pattern of *spp1* and show co-localisation with cardiac macrophages and fibroblasts.** (A-C) Lateral images of the head of a 5 dpf TgBAC(*spp1:mCherry*) fish labelled with *in situ* hybridisation for *spp1* (A) and immunostaining for RFP expression (B,C) demonstrating co-localisation in the developing craniofacial skeleton, specifically the operculum and cleithrum as previously described ^1-3^. Anterior is to the left. Op = Operculum, Cl = Cleithrum. (D-F) Single z-plane confocal images of the injured region of the heart of a TgBAC(*spp1:mCherry*) fish at 3 dpi labelled with *in situ* hybridisation for *spp1* (D) and immunostaining for RFP expression (E,F). (G) 3D reconstruction of the injured region of a TgBAC(*spp1:mCherry*); *ET37* heart at 3 dpi. Co-localisation is observed in a subset of GFP+ fibroblasts (arrowed). (H,I) Representative maximum projection images of TgBAC(*spp1:mCherry*); TgBAC(*tnfα:GFP*) fish at 3 and 7 dpi. Double (arrowed) and single *spp1+* positive cells (asterisks) are observed at both time-points. 3D projections were also checked to confirm co-localisation. Scale bars: A-C = 100 μm; D-G = 50 μm; H,I = 20 μm.

**
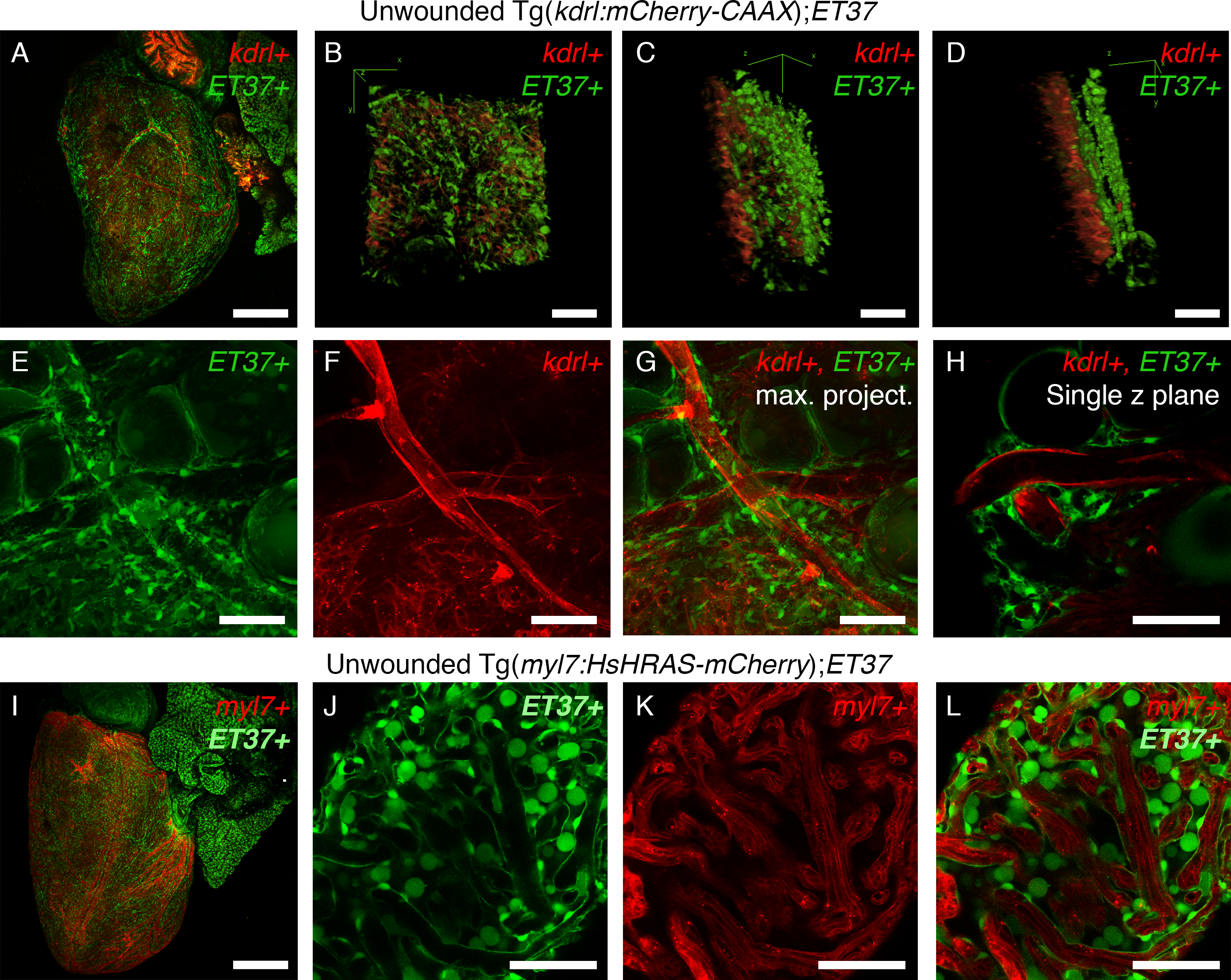
**

**Figure S7 – *ET37* transgenic fish label interstitial cells in the heart.** (A-H) Confocal imaging of unwounded Tg(*kdrl:mCherry-CAAX*); *ET37* fish hearts. (A-D) View of a whole heart (A) or 3D projection views of the surface of the unwounded ventricle demonstrate a layer of GFP+ cells encasing the main vasculature of the myocardium (B-D). (E-H) High magnification, maximum projection (E-G) or single z-plane (H) views of the surface of the ventricle demonstrating a population of GFP+ cells surrounding the coronary arteries which do not co-localise with RFP+ endothelial cells. We cannot rule out the possibility that ET37 labels pericytes and/or adipocytes as well as fibroblast-like interstitial cells. (I-J) Confocal and multi-photon imaging of unwounded Tg(*myl7:HsHRAS-mCherry*); *ET37* fish hearts. View of a whole heart (I) and single z-plane images (J-L) deeper in the myocardium reveal GFP+ cells interspersed between mCherry+ cardiomyocytes. Max project. = maximum projection. Scale bars: A,I = 250 μm; B-H,J-L = 50 μm.

**
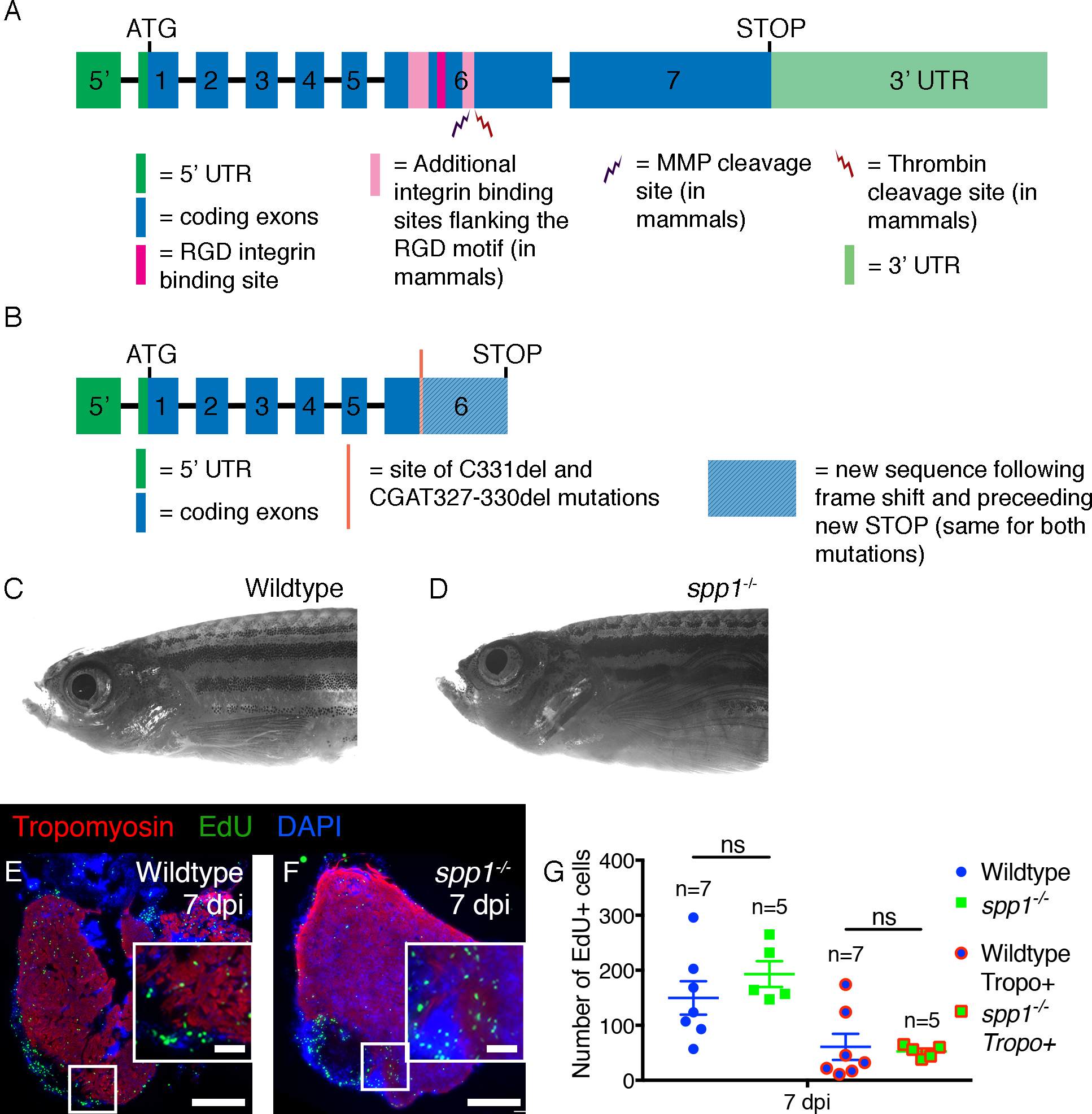
**

**Figure S8 – Details of the mutations in *spp1* and description of the overall phenotype. Proliferation is unaltered in *spp1^-/-^* fish.** (A) Schematic of the zebrafish *spp1* gene structure. The ATG and STOP codons are indicated as well as the position of the RGD integrin binding site (conserved between zebrafish and mammals). The position of additional integrin binding sites and extracellular cleavage sites known from mammals are also indicated. Their positions or existence are not known in zebrafish, so their positions are predicted based on the mammalian position in relation to the RGD. All exons are to scale, introns are not to scale. (B) Schematic showing the position and the effect of the C331del and CGAT327-330del mutations in zebrafish *spp1*. Both result in frameshifts and a premature STOP codon. (C,D) Representative images of an adult wildtype zebrafish (C) and a *spp1*^-/-^ adult mutant (D). These fish appear grossly normal as adults. (E-G) Representative images and quantification (G) of the number of EdU+, proliferative cells in wildtype (E) and *spp1^-/-^*  fish (F) at 7 dpi. The number of Tropomyosin+, EdU+ positive cardiomyocytes were also quantified (G). In G, statistical analysis was performed by Mann-Whitney tests of control and *spp1*^-/-^ data. Scale bars: E,F = 250 μm; inset in E,F = 50 μm.

**Supplementary Methods**

**Zebrafish lines and cardiac injury**

The Tg(*mpx:GFP*)i114 ^4^, Tg(*lyz:dsRED2*) ^5^, Tg(*gata2a:eGFP*) ^6^, Tg(*mpeg1:mCherry*) ^7^, Tg(*c-fms:GFP*) ^8^, Tg(*lck:GFP*) ^9^, TgBAC(*tnfα:GFP*) ^10^, *csf1ra^j4e1/j4e1^* ^11^, *ET37* ^12^, Tg(*kdrl:mCherry-CAAX*) ^13^ and Tg(*myl7:HsHRAS-mCherry*) ^14^ fish have been described previously. Fish were randomly assigned to control or test experiments using a random number generator. Whenever possible fish from the same tank were used for each experiment and each experiment repeated at least twice with fish from different tanks to control for natural variation. For mutant analyses, siblings were used as controls. Cardiac injuries were carried out as described previously ^15^. Briefly, fish were anaesthetised in 0.13% MS-222 (Sigma; A5040) and placed ventral side up in a pre-cut sponge soaked in aquarium water containing anaesthetic. A 4 mm incision was made through the skin and the pericardial sac directly above the heart to expose the ventricle. The ventricle was dried using a sterile cotton swab and a liquid nitrogen cooled probe applied to the exposed ventricle for 30 seconds. Fish used were aged 4 - 18 months. Sham injury involved opening the skin and pericardial sac but not damaging the heart.

**Immunofluorescence analysis, histology, *in situ* hybridisation and imaging**

Whole, dissected hearts from transgenic zebrafish were fixed in 4% paraformaldehyde (PFA) overnight at 4^o^C, washed twice in PBS and mounted in 1.5% low melting point agarose. For whole-mount immunostaining, cardiac tissue was fixed as above, blocked in 10% goat serum for 1 hour, incubated in primary antibody overnight at 4^o^C, washed extensively in PBS containing 0.5% Triton-X, incubated in secondary antibody overnight at 4^o^C, washed extensively and post-fixed in 4% PFA. Imaging was performed on a Leica SP8 confocal scanning microscope or Leica SP8 tandem scanning system with Spectra Physics Deep See (680-1300nm plus 1040nm) laser for multiphoton excitation. For immunostaining of sections, hearts were embedded in paraffin wax, sectioned and stained as described previously ^16^. Images were acquired on a Leica DMI6000 widefield microscope. Antibodies used were: anti-Collagen I (1:200, Abcam, ab23730), anti-L-plastin (1:500; ^17,18^), Alexa Fluor 546 (1:500; Life technologies; A11035) and Alexa Fluor 647 (1:200; Life technologies; A21449). For proliferation analysis, 10 mM EdU was IP injected into cryoinjured fish 24 hours prior to termination. Dissected hearts were fixed as described above, cryopreserved in 30% sucrose and frozen embedded in OCT. EdU labelling was performed on cryosections according to the manufacturers protocol (Molecular Probes, C10340) and co-stained with an anti-tropomyosin antibody (1:50; The Developmental Studies Hybridoma Bank, University of Iowa). Quantification is presented as the average total number of EdU+ cells per ventricle across three sections through the injury. AFOG staining on paraffin embedded sections was performed by standard histological procedures.

**Supplementary References**

1 Laue, K., Janicke, M., Plaster, N., Sonntag, C. & Hammerschmidt, M. Restriction of retinoic acid activity by Cyp26b1 is required for proper timing and patterning of osteogenesis during zebrafish development. *Development* **135**, 3775-3787, doi:10.1242/dev.021238 (2008).

2 Torregroza, I. *et al.* Regulation of a vascular plexus by gata4 is mediated in zebrafish through the chemokine sdf1a. *PloS one* **7**, e46844, doi:10.1371/journal.pone.0046844 (2012).

3 Apschner, A., Huitema, L. F., Ponsioen, B., Peterson-Maduro, J. & Schulte-Merker, S. Zebrafish enpp1 mutants exhibit pathological mineralization, mimicking features of generalized arterial calcification of infancy (GACI) and pseudoxanthoma elasticum (PXE). *Disease models & mechanisms* **7**, 811-822, doi:10.1242/dmm.015693 (2014).

4 Renshaw, S. A. *et al.* A transgenic zebrafish model of neutrophilic inflammation. *Blood* **108**, 3976-3978, doi:10.1182/blood-2006-05-024075 (2006).

5 Hall, C., Flores, M. V., Storm, T., Crosier, K. & Crosier, P. The zebrafish lysozyme C promoter drives myeloid-specific expression in transgenic fish. *BMC developmental biology* **7**, 42, doi:10.1186/1471-213x-7-42 (2007).

6 Traver, D. *et al.* Transplantation and in vivo imaging of multilineage engraftment in zebrafish bloodless mutants. *Nature immunology* **4**, 1238-1246, doi:10.1038/ni1007 (2003).

7 Ellett, F., Pase, L., Hayman, J. W., Andrianopoulos, A. & Lieschke, G. J. mpeg1 promoter transgenes direct macrophage-lineage expression in zebrafish. *Blood* **117**, e49-56, doi:10.1182/blood-2010-10-314120 (2011).

8 Dee, C. T. *et al.* CD4-Transgenic Zebrafish Reveal Tissue-Resident Th2- and Regulatory T Cell-like Populations and Diverse Mononuclear Phagocytes. *Journal of immunology (Baltimore, Md. : 1950)* **197**, 3520-3530, doi:10.4049/jimmunol.1600959 (2016).

9 Langenau, D. M. *et al.* In vivo tracking of T cell development, ablation, and engraftment in transgenic zebrafish. *Proceedings of the National Academy of Sciences of the United States of America* **101**, 7369-7374, doi:10.1073/pnas.0402248101 (2004).

10 Marjoram, L. *et al.* Epigenetic control of intestinal barrier function and inflammation in zebrafish. *Proceedings of the National Academy of Sciences of the United States of America* **112**, 2770-2775, doi:10.1073/pnas.1424089112 (2015).

11 Parichy, D. M., Ransom, D. G., Paw, B., Zon, L. I. & Johnson, S. L. An orthologue of the kit-related gene fms is required for development of neural crest-derived xanthophores and a subpopulation of adult melanocytes in the zebrafish, Danio rerio. *Development* **127**, 3031-3044 (2000).

12 Choo, B. G. *et al.* Zebrafish transgenic Enhancer TRAP line database (ZETRAP). *BMC developmental biology* **6**, 5, doi:10.1186/1471-213x-6-5 (2006).

13 Fujita, M. *et al.* Assembly and patterning of the vascular network of the vertebrate hindbrain. *Development* **138**, 1705-1715, doi:10.1242/dev.058776 (2011).

14 Yoruk, B., Gillers, B. S., Chi, N. C. & Scott, I. C. Ccm3 functions in a manner distinct from Ccm1 and Ccm2 in a zebrafish model of CCM vascular disease. *Developmental biology* **362**, 121-131, doi:10.1016/j.ydbio.2011.12.006 (2012).

15 Gonzalez-Rosa, J. M. & Mercader, N. Cryoinjury as a myocardial infarction model for the study of cardiac regeneration in the zebrafish. *Nature protocols* **7**, 782-788, doi:10.1038/nprot.2012.025 (2012).

16 Richardson, R. *et al.* Adult zebrafish as a model system for cutaneous wound-healing research. *The Journal of investigative dermatology* **133**, 1655-1665, doi:10.1038/jid.2013.16 (2013).

17 Cvejic, A. *et al.* Analysis of WASp function during the wound inflammatory response--live-imaging studies in zebrafish larvae. *Journal of cell science* **121**, 3196-3206, doi:10.1242/jcs.032235 (2008).

18 Feng, Y., Santoriello, C., Mione, M., Hurlstone, A. & Martin, P. Live imaging of innate immune cell sensing of transformed cells in zebrafish larvae: parallels between tumor initiation and wound inflammation. *PLoS biology* **8**, e1000562, doi:10.1371/journal.pbio.1000562 (2010).

19 Bussmann, J. & Schulte-Merker, S. Rapid BAC selection for tol2-mediated transgenesis in zebrafish. *Development* **138**, 4327-4332, doi:10.1242/dev.068080 (2011).

20 Chablais, F., Veit, J., Rainer, G. & Jazwinska, A. The zebrafish heart regenerates after cryoinjury-induced myocardial infarction. *BMC developmental biology* **11**, 21, doi:10.1186/1471-213x-11-21 (2011).
